# Identification of multiple male reproductive tract-specific proteins that regulate sperm migration through the oviduct in mice

**DOI:** 10.1073/pnas.1908736116

**Published:** 2019-08-27

**Authors:** Yoshitaka Fujihara, Taichi Noda, Kiyonori Kobayashi, Asami Oji, Sumire Kobayashi, Takafumi Matsumura, Tamara Larasati, Seiya Oura, Kanako Kojima-Kita, Zhifeng Yu, Martin M. Matzuk, Masahito Ikawa

**Affiliations:** ^a^Research Institute for Microbial Diseases, Osaka University, Suita, 565-0871 Osaka, Japan;; ^b^Graduate School of Pharmaceutical Sciences, Osaka University, Suita, 565-0871 Osaka, Japan;; ^c^Center for Drug Discovery, Baylor College of Medicine, Houston, TX 77030;; ^d^Department of Pathology & Immunology, Baylor College of Medicine, Houston, TX 77030;; ^e^Department of Bioscience and Genetics, National Cerebral and Cardiovascular Center, Suita, 564-8565 Osaka, Japan;; ^f^Graduate School of Frontier Biosciences, Osaka University, Suita, 565-0871 Osaka, Japan;; ^g^Graduate School of Medicine, Osaka University, Suita, 565-0871 Osaka, Japan;; ^h^The Institute of Medical Science, The University of Tokyo, Minato-ku, 108-8639 Tokyo, Japan

**Keywords:** CRISPR/Cas9, fertilization, infertility, transgenic, uterotubal junction

## Abstract

CRISPR/Cas9-mediated genome editing technology enables researchers to efficiently generate and analyze genetically modified animals. We have taken advantage of this game-changing technology to uncover essential factors for fertility. In this study, we generated knockouts (KOs) of multiple male reproductive organ-specific genes and performed phenotypic screening of these null mutant mice to attempt to identify proteins essential for male fertility. We focused on making large deletions (dels) within 2 gene clusters encoding cystatin (CST) and prostate and testis expressed (PATE) proteins and individual gene mutations in 2 other gene families encoding glycerophosphodiester phosphodiesterase domain (GDPD) containing and lymphocyte antigen 6 (Ly6)/Plaur domain (LYPD) containing proteins. These gene families were chosen because many of the genes demonstrate male reproductive tract-specific expression. Although *Gdpd1* and *Gdpd4* mutant mice were fertile, disruptions of *Cst* and *Pate* gene clusters and *Lypd4* resulted in male sterility or severe fertility defects secondary to impaired sperm migration through the oviduct. While absence of the epididymal protein families CST and PATE affect the localization of the sperm membrane protein A disintegrin and metallopeptidase domain 3 (ADAM3), the sperm acrosomal membrane protein LYPD4 regulates sperm fertilizing ability via an ADAM3-independent pathway. Thus, use of CRISPR/Cas9 technologies has allowed us to quickly rule in and rule out proteins required for male fertility and expand our list of male-specific proteins that function in sperm migration through the oviduct.

## Significance

While the emergence of gene modification technologies has produced major discoveries in biomedical sciences, the recent development of the CRISPR/Cas9 system has dramatically altered the trajectory of phenotypic analysis in animal models. In this study, we identified male-specific gene clusters (*Cst* and *Pate*) and family genes (*Gdpd* and *Lypd*) and found specific members to be required for male fertility, especially for sperm fertilizing ability. Our findings support the important roles of these proteins in sperm function and could be used to develop novel infertility treatments as well as contraceptives.

We established a quick and efficient system to analyze male fertility in vivo using CRISPR/Cas9-mediated mutant mice (1, 2). With this system in place, we found that 93 evolutionarily conserved and reproductive tract-enriched genes are not individually essential for male fertility in mice (3–5). Moreover, we suggest that researchers should determine whether a gene of interest is required for male fertility in vivo before spending significant effort to analyze the molecular function of the gene in vitro. In parallel, we also established an efficient method of genome editing in the mouse embryonic stem (ES) cells. This method can easily introduce large genomic dels (more than 100 kb) in ES cells with 2 single guide RNAs (sgRNAs) (6, 7). Deleting large regions of gene clusters has an advantage to avoid problems with functional compensation by similar proteins encoded by the same cluster genes. Furthermore, through the use of high-throughput screening of KO mice, we can find new targets for contraceptive development. In the present study, we focused on analyzing 2 gene clusters [*Cst* and *Pate*] and 2 gene families [*Gdpd* and *Lypd*] in mice. To examine the physiological roles of these male reproductive tract-specific (testis [Te] and epididymis [Epi]) genes, we produced genetically modified mice by the CRISPR/Cas9 system and conventional methods.

Fourteen *Pate* family genes form a cluster on mouse chromosome 9, and almost all of these genes are strongly expressed in the prostate (Pr) and Te in mice (8). Another study showed that these genes are abundantly expressed in the Epi (9). *Pate* family genes encode proteins with a signal peptide and a consensus sequence pattern of 10 cysteines (8, 10). This specific pattern of cysteines is found in a large family of three-fingered proteins, such as the urokinase plasminogen activator receptor (uPAR) and the LY6 family (11, 12). Ly6/uPAR family members are classified as membrane-tethered or secreted proteins based on their subcellular localization (10). Previous papers showed that secreted Ly6/uPAR proteins including *Pate* family genes may function as regulators/modulators for receptors including the nicotinic acetylcholine receptor (8, 10). Recently, we revealed that *Pate1*, *Pate2*, or *Pate3* single mutant males are fertile (3) but that *Pate4* KO males are subfertile due to plug formation defects (13). The results indicate that some, not all, but *Pate* family genes are required for male fertility.

The CST family is secreted cysteine protease inhibitors that share a CST-like domain and have two disulfide bonds located near the carboxyl terminus (14). CST3 is well known for its functions in the nervous system, cardiovascular system, and kidney (Ki) (15, 16). Previous studies have shown that 8 genes (*Cstl1*-*Cstdc2*) are located contiguously with *Cst3* on chromosome 2 and are strongly expressed in the male reproductive tract (17–19). Although CSTs are involved in the formation of the amyloid matrix that appears to function in maturation of spermatozoa in the epididymal lumen (20, 21), *Cst8* or *Cst9* single mutant male mice are fertile (22, 23). In this study, we generated mice lacking the entire male reproductive tract-specific CST family and examined the phenotypes of these cluster del mice.

Glycosylphosphatidylinositol-anchored proteins (GPI-APs) are anchored to the outer cell membrane by GPI and are critical for physiological events, such as development, immunity, neurogenesis, and fertilization (24). We reported previously that the testicular germ cell (TGC)-specific GPI-AP complex TEX101/LY6K is required for sperm migration through the oviduct and male fertility (25). Also, post-GPI attachment to proteins 1 (PGAP1) is a GPI inositoldeacylase that removes the palmitate from inositol and is essential for male fertility (26). These reports indicate that there is a strong correlation between GPI-APs and sperm fertilizing ability (27). Moreover, both membrane-anchored and soluble GPI-APs play roles in regulating protein conformation and functional properties (24). In the present study, we examined the functions of Te-enriched protein LYPD4 from the LYPD GPI-AP family and GDPD1 and GDPD4 from the GDPD family of putative GPI-AP-releasing (GPIase) enzymes. Since their physiological roles remain to be determined in vivo, we generated KO mice and analyzed their fertility.

Our conclusions from these studies are that male mice lacking two gene clusters (*Cst* and *Pate*) and *Lypd4* demonstrated severe fertility defects due to impaired sperm migration through the oviduct and impaired sperm binding ability to zona pellucida (ZP) in vitro. These phenotypes are shared among mice lacking sperm membrane protein ADAM3. ADAM3 is thought to play a pivotal role in sperm-ZP binding and sperm migration through the uterotubal junction (UTJ) (28, 29). More than 10 proteins (ACE, ADAM1A, ADAM2, CALR3, CLGN, CMTM2A/B, PDILT, PMIS2, PRSS37, RNASE10, TEX101, and TPST2) have been described that affect the ADAM3 protein and/or its processing/localization in spermatozoa (27, 30). We also discovered that the TEX101/LY6K protein complex interacts transiently with the membrane protein ADAM3 (31). Although ADAM3 disappeared from spermatozoa in *Cst* and *Pate* cluster del mice, ADAM3 remained in *Lypd4* KO spermatozoa, similar to *Ly6k* and *Pgap1* KO mice (25, 26).

## Results

### Expression Patterns of *Pate* Family Genes.

**Fig. 1.**
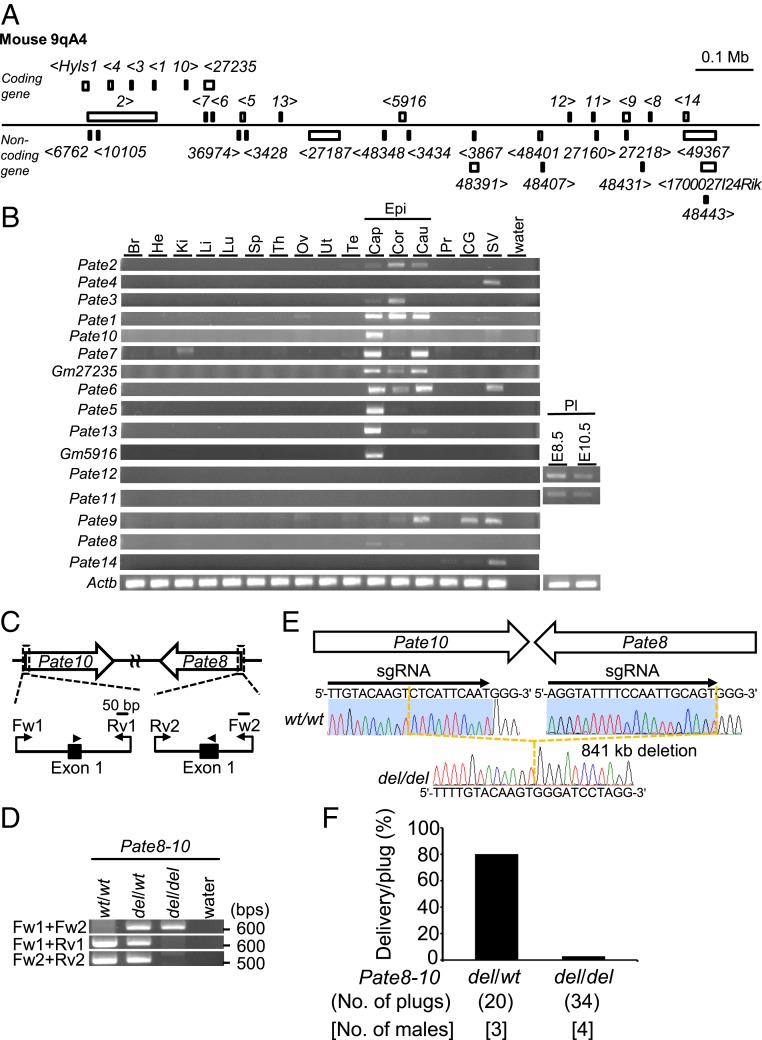
Male fertility of mice lacking the region between *Pate8* and *Pate10*. (*A*) *Pate* family genes within murine genomic locus |chromosome 9qA4|. *Pate* family genes (*Pate1*–*Pate14*) were shown as “1” to “14.” Genes with “Gm-” were listed as serial numbers. The inequalities showed the direction of transcription. (*B*) Multitissue gene expression by RT-PCR analysis. Actin β (*Actb*) was used as the control. Brain (Br), heart (He), kidney (Ki), liver (Li), lung (Lu), spleen (Sp), thymus (Th), ovary (Ov), uterus (Ut), testis (Te), epididymis (Epi), caput (Cap), corpus (Cor), cauda (Cau), prostate (Pr), coagulating gland (CG), seminal vesicle (SV), placenta (Pl). (*C*) gRNA design for del of gene cluster between *Pate8* and *Pate10*. Arrowheads show gRNAs for *Pate8* and *Pate10*. Primers (Fw1, Fw2, Rv1, and Rv2) were used for genotyping with PCR. (*D*) Genotyping with PCR in (*Pate8*–*Pate10*)^*del/del*^ mice. Primers shown in *C* were used for PCR. wild-type (WT). (*E*) DNA sequencing. The sequence of PCR amplicon was analyzed. (*Pate8*–*Pate10*)^*del/del*^ mice deleted 841-kb genomic region between *Pate8* and *Pate10*. (*F*) Pregnancy rates (delivery/plug). Males were caged with two WT females. (*Pate8*–*Pate10*)^*del/del*^ males succeeded in the mating, but the pregnancy rates of these females significantly reduced (*del/wt*: 80% [16/20], *del/del*: 2.9% [1/34]).

On the mouse chromosome 9qA4 locus, we found a gene cluster containing 14 *Pate* family genes, 2 coding genes (*Gm27235* and *Gm5916*), and 17 noncoding genes (Fig. 1*A*). The *Pate* family specific patterns of cysteine residues are conserved in GM27235 and GM5916 suggesting these are novel PATE family members (*SI Appendix*, Fig. S1*A*). We examined the expression patterns of these coding genes by RT-PCR with mouse multiple tissue. Most of the *Pate* family genes (*Pate1*–*3*, *Pate5*–*10*, and *Pate13*), *Gm27235*, and *Gm5916* were expressed in the Epi rather than the Te and Pr. The remaining genes showed different tissue-specific gene expression patterns (*Pate4* and *14* in seminal vesicles [SVs] and *Pate11* and *12* in placenta [Pl]) (Fig. 1*B*). The result of RT-PCR analysis was confirmed by the RNA-sequencing (RNA-seq) data (*SI Appendix*, Table S1). Our results correlate with a previous report indicating that most *Pate* family genes are abundantly expressed in the Epi (9).

### Fertility of Mice Lacking *Pate* Family Genes.

**Fig. 2.**
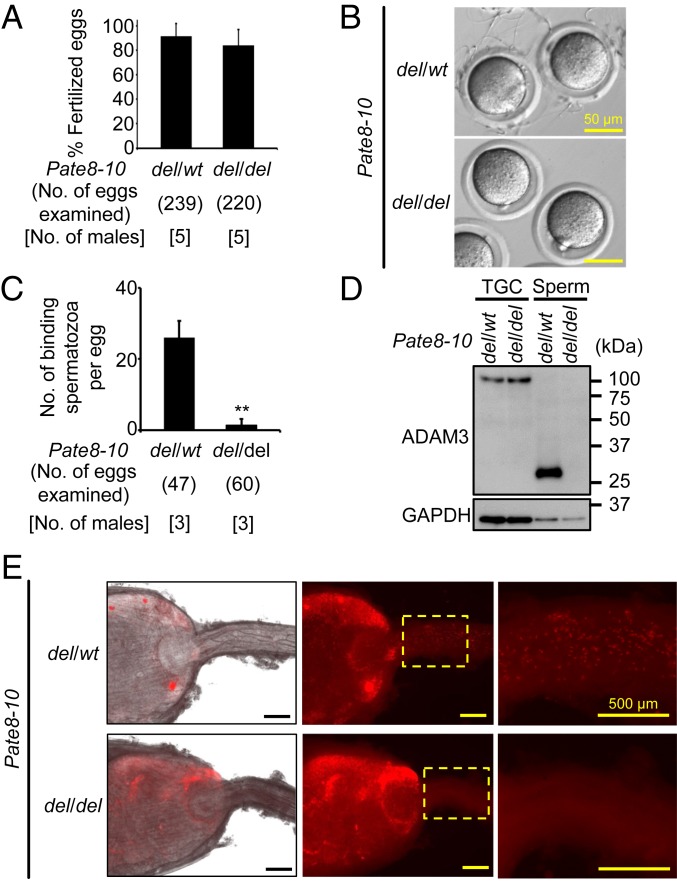
Phenotypic analysis of (*Pate8*–*Pate10*)^*del/del*^ male mice. (*A*) Sperm fertilizing ability of using cumulus-intact oocytes in vitro. (*Pate8*–*Pate10*)^*del/del*^ spermatozoa could efficiently fertilize eggs (*del/wt*: 91.4 ± 10.5%, *del/del*: 84.0 ± 13.0%; mean ± SD). (*B*) Sperm ZP-binding assay. Spermatozoa were inseminated with cumulus-free oocytes, but (*Pate8*–*Pate10*)^*del/del*^ spermatozoa hardly bind to the ZP. (*C*) Average number of spermatozoa bound to the ZP. The number of spermatozoa bound to the ZP in (*Pate8*–*Pate10*)^*del/del*^ males was significantly reduced (*del/wt*: 25.9 ± 4.7 spermatozoa/egg, *del/del*: 1.5 ± 1.6 spermatozoa/egg; mean ± SD). **P < 0.01, Student’s *t* test. (*D*) Detection of ADAM3. ADAM3 was not detected in (*Pate10*–*Pate8*)^*del/del*^ spermatozoa (also see *SI Appendix*, Fig. S4*B*). GAPDH was used as the control. Sperm: Cau Epi spermatozoa. (*E*) Observation of sperm migration into the female reproductive tract. For sperm observation in the female reproductive tract after mating, we crossed (*Pate8*–*Pate10*)^*del/del*^ mice with a transgenic mouse line in which the acrosome and mitochondria can be visualized by EGFP and DsRed2, respectively. (*Pate8*–*Pate10*)^*del/del*^ spermatozoa could not pass through the UTJ.

We previously described that *Pate1*–*Pate3* single mutant males are fertile (3), but the remaining *Pate* family genes may compensate for the physiological function in single mutant males. Thus, we produced mice lacking the region between *Pate1* and *Pate3* which is conserved in humans and mice (*SI Appendix*, Fig. S1 *A* and *B*). We obtained a heterozygous mutant mouse by introducing two gRNAs and the CAS9 enzyme for this large del. (*Pate1*–*Pate3*)^*del/del*^ mice were obtained by heterozygous F1 intercrosses (*SI Appendix*, Fig. S1 *C* and *D*). The pregnancy rate (delivery per plug) of females mated with (*Pate1*–*Pate3*)^*del/del*^ males was comparable to controls (*SI Appendix*, Fig. S1*E*). The average litter size was 9.1 ± 0.6 for female mice mated with control males (*n* = 3) and 10.4 ± 0.5 for females mated with (*Pate1*–*Pate3*)^*del/del*^ males (*n* = 3). There was no apparent difference between (*Pate1*–*Pate3*)^*del/del*^ and control males in the histology of Epi, sperm morphology, and motility parameters (*SI Appendix*, Fig. S2). Thus, *Pate1*–*3* are not essential for male fertility in mice. Next, we attempted to make mice lacking the region between *Pate8* and *Pate10*, which is not conserved in humans (Fig. 1*C* and *SI Appendix*, Fig. S1*A*). We obtained chimeric mice by injecting ES cells with a heterozygous mutant for this large del. (*Pate8*–*Pate10*)^*del/del*^ mice were obtained by heterozygous F1 intercrosses. The del was confirmed by PCR (Fig. 1*D*) and direct sequencing (Fig. 1*E*). (*Pate8*–*Pate10*)^*del/del*^ females were fertile, but (*Pate8*–*Pate10*)^*del/del*^ males showed severe subfertility (Fig. 1*F*). The average litter size was 9.6 ± 0.3 for female mice mated with heterozygous mutant males (*n* = 3), and 0.3 ± 0.5 for females mated with (*Pate8*–*Pate10*)^*del/del*^ males (*n* = 4). There was no apparent difference between (*Pate8*–*Pate10*)^*del/del*^ and control males in the histology of Epi, sperm morphology, and motility parameters (*SI Appendix*, Fig. S3). (*Pate8*–*Pate10*)^*del/del*^ spermatozoa could fertilize cumulus-intact oocytes (Fig. 2*A*). However, (*Pate8*–*Pate10*)^*del/del*^ spermatozoa barely bound the ZP, resulting in the lowered fertilization rates with cumulus-free oocytes (Fig. 2 *B* and *C* and *SI Appendix*, Fig. S4*A*). There was no apparent difference in the characterization of ADAM3 in TGC between (*Pate8*– *Pate10*)^*del/del*^ and control males, but the signal disappeared in (*Pate8*–*Pate10*)^*del/del*^ males during Epi sperm maturation (Fig. 2*D* and *SI Appendix*, Fig. S4*B*). To observe the sperm behavior in the female reproductive tract, we crossed (*Pate8*–*Pate10*)^*del/del*^ mice with a transgenic mouse line in which the acrosome and mitochondria can be visualized by EGFP and DsRed2, respectively. When we observed the ejaculated spermatozoa through the wall of the female reproductive tract, we found almost equal amounts of spermatozoa in the uterus (Ut) of wild-type (WT) females mated with WT males compared to (*Pate8*–*Pate10*)^*del/del*^ males. However, (*Pate8*–*Pate10*)^*del/del*^ spermatozoa had a disruption in migration through the UTJ (Fig. 2*E*). Thus, (*Pate8*–*Pate10*)^*del/del*^ males are severely subfertile due to lack of ADAM3 from mature spermatozoa and subsequent defects in sperm migration through the UTJ.

### Male Reproductive Tract-Specific *Cst* Family Genes.

**Fig. 3.**
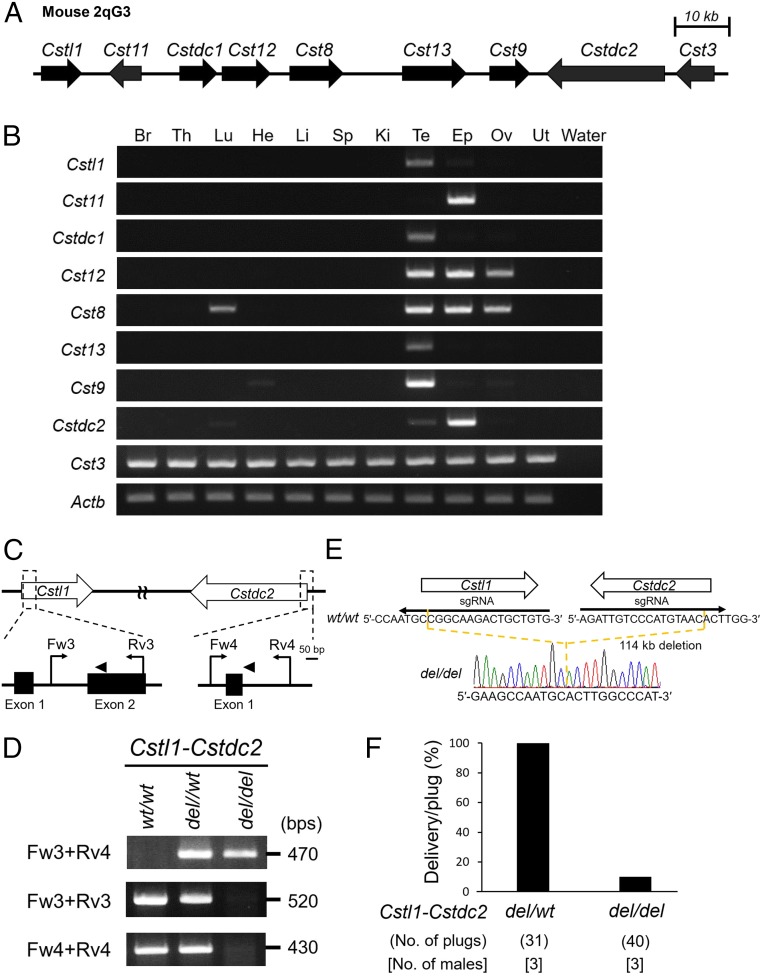
Male fertility of mice lacking the region between *Cstl1* and *Cstdc2*. (*A*) Male reproductive CST family within murine genomic location |chromosome 2qG3|. The direction of the arrow indicates the direction of the transcription. (*B*) Multitissue gene expression by RT-PCR analysis. *Actb* was used as an expression control. (*C*) gRNA design for del of gene cluster between *Cstl1* and *Cstdc2*. Arrowheads show gRNAs for *Cstl1* and *Cstdc2*. Primers (Fw3, Fw4, Rv3, and Rv4) were used for genotyping with PCR. (*D*) Genotyping with PCR in (*Cstl1-Cstdc2*)^*del/del*^ mice. Primers shown in *C* were used for PCR. (*E*) DNA sequencing. The sequence of PCR amplicon was analyzed. (*Cstl1-Cstdc2*)^*del/del*^ mice deleted 114-kb genomic region between *Cstl1* and *Cstdc2*. (*F*) Pregnancy rates (%) of WT female mice mated with (*Cstl1-Cstdc2*) mutant male mice. The average pregnancy rate (delivery per plug) of females coupled with (*Cstl1-Cstdc2*)^*del/wt*^ and (*Cstl1-Cstdc2*)^*del/del*^ male mice were 100% (31/31) and 10.0% (4/40), respectively.

Nine genes related to the CST family are located continuously on mouse chromosome 2qG3 locus (Fig. 3*A*). We examined the expression pattern of these genes by RT-PCR with mouse multiple tissue. *Cstl1*, *Cstdc1*, *Cst9*, and *Cst13* are strongly expressed in Te, *Cstdc2* and *Cst11* were strongly expressed in the Epi, and *Cst8* and *Cst12* were abundantly expressed in the Te, Epi, and ovary (Ov). *Cst3* was expressed ubiquitously (Fig. 3*B*). The RT-PCR results were confirmed by the RNA-seq data (*SI Appendix*, Table S1). These data show that 8 of these 9 genes are specifically expressed in the reproductive tract and could suggest a function of these genes in male reproduction.

### Phenotype of Mice Lacking the Reproductive Tract-Specific *Cst* Family Genes.

**Fig. 4.**
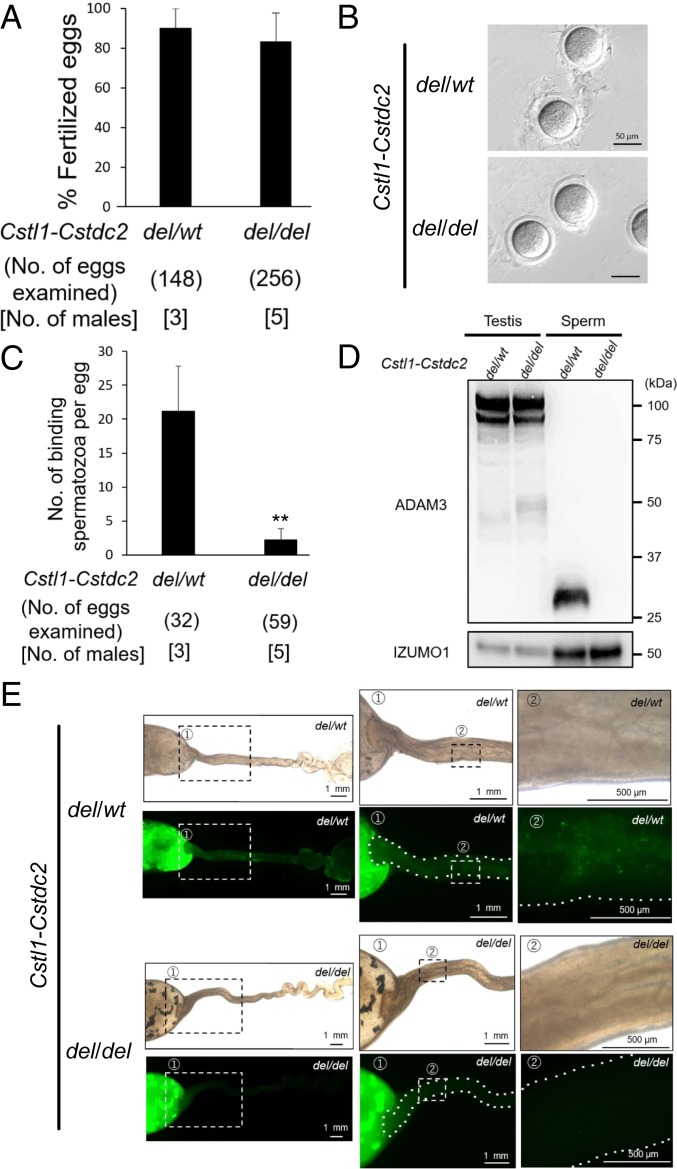
Phenotypic analysis of (*Cstl1-Cstdc2*)^*del/del*^ male mice. (*A*) In vitro fertilization rates using (*Cstl1-Cstdc2*) mutant spermatozoa. Average fertilization rates of (*Cstl1-Cstdc2*)^*del/wt*^ and (*Cstl1-Cstdc2*)^*del/del*^ spermatozoa were 90.4 ± 9.6% (136/148 eggs) and 83.4 ± 14.3% (222/256 eggs), respectively. (*B*) Sperm-ZP-binding assay. Spermatozoa were inseminated with cumulus-free oocytes, but (*Cstl1*-*Cstdc2*)^*del/del*^ spermatozoa hardly bind to the ZP. (*C*) Average number of ZP-binding spermatozoa in vitro. The number of ZP-binding spermatozoa in (*Cstl1*-*Cstdc2*)^*del/del*^ mice (2.2 ± 1.6 spermatozoa/egg; mean ± SD) was significantly reduced compared with that of (*Cstl1-Cstdc2*)^*del/wt*^ mice (21.2 ± 6.7 spermatozoa/egg). **P < 0.01, Student’s *t* test. (*D*) Immunoblot analysis of ADAM3. ADAM3 was not detectable in (*Cstl1*-*Cstdc2*)^*del/del*^ spermatozoa. IZUMO1 was used as the control. Sperm: Cau Epi spermatozoa. (*E*) Observation of sperm migration into the female reproductive tract. For sperm observation in the female reproductive tract after mating, we crossed (*Cstl1*-*Cstdc2*)^*del/del*^ mice with a transgenic mouse line in which the acrosome can be visualized by EGFP. (*Cstl1*-*Cstdc2*)^*del/del*^ spermatozoa could not pass through the UTJ.

To study the functions of the *Cst* family, we generated a del of the family between *Cstl1* and *Cstdc2* in ES cells by transfecting 2 sgRNA/Cas9 coexpressing plasmids (Fig. 3*C*), generated chimeric mice from *Cst-*del ES cells, and obtained (*Cstl1-Cstdc2*)^*del/del*^ mice from heterozygous F1 intercrosses. (*Cstl1-Cstdc2*)^*del/del*^ mice were confirmed by genomic PCR (Fig. 3*D*) and direct sequencing (Fig. 3*E*). There was no difference in Te morphology and histology and sperm morphology and motility between (*Cstl1-Cstdc2*)^*del/wt*^ and (*Cstl1-Cstdc2*)^*del/del*^ mutant mice (*SI Appendix*, Figs. S5 and S6 *A* and *B*). To evaluate male fertility, we checked the pregnancy rate and the litter size in (*Cstl1-Cstdc2*)^*del/wt*^ and (*Cstl1-Cstdc2*)^*del/del*^ mutant mice. The pregnancy rate (delivery per plug) was 100% (31/31 plugs) of female mice mated with (*Cstl1-Cstdc2*)^*del/wt*^ males but only 10.0% (4/40 plugs) of females mated with (*Cstl1-Cstdc2*)^*del/del*^ males (Fig. 3*F*). The average litter size was 9.2 ± 0.8 for female mice mated with (*Cstl1-Cstdc2*)^*del/wt*^ males (*n* = 3), and 1.8 ± 1.5 for females mated with (*Cstl1-Cstdc2*)^*del*/*del*^ males (*n* = 3). To determine the cause of the subfertility, we performed in vitro fertilization (IVF). (*Cstl1-Cstdc2*)^*del/del*^ cauda (Cau) epididymal spermatozoa could fertilize cumulus-intact oocytes by IVF (Fig. 4*A*). Next, we examined sperm ZP-binding ability with cumulus-free oocytes. (*Cstl1-Cstdc2*)^*del/del*^ Cau epididymal spermatozoa barely bind the ZP (Fig. 4 *B* and *C*). Based on this result, we performed IVF using cumulus-free oocytes. Fertilization rate of (*Cstl1-Cstdc2*)^*del/del*^ spermatozoa decreased considerably compared to heterozygous mutant spermatozoa (*SI Appendix*, Fig. S6*C*). Because these phenotypes are similar to those of *Adam3* KO mice, we checked the ADAM3 protein by immunoblot analysis. Whereas there was no difference in ADAM3 Te protein levels between (*Cstl1-Cstdc2*)^*del/wt*^ and (*Cstl1-Cstdc2*)^*del/del*^ males, the signal disappeared in (*Cstl1-Cstdc2*)^*del/del*^ Cau Epi spermatozoa (Fig. 4*D*). Moreover, we found that ADAM3 disappeared from Cap Epi spermatozoa in (*Cstl1-Cstdc2*)^*del/del*^ mice (*SI Appendix*, Fig. S6*D*). As expected, (*Cstl1-Cstdc2*)^*del/del*^ spermatozoa failed to migrate from the Ut to the oviduct (Fig. 4*E*). These results indicate that male reproductive CST family is required for ADAM3 stability and for efficient migration of mouse spermatozoa from the Ut to the oviduct.

### Te-Specific Expression of *Gdpd* Family Genes and Male Fertility of *Gdpd1* and *Gdpd4* Mutant Mice.

In mice, the *Gdpd* family consists of 5 genes (*Gdpd1-5*). The expression of these genes in various mouse tissues was examined by RT-PCR. *Gdpd1*, *Gdpd4*, and *Gdpd5* are expressed strongly in the Te (*SI Appendix*, Fig. S7*A*). *Gdpd5* (previously named *Gde2*) KO mice have been reported previously, and GDPD5 is a key regulator of motor neuron differentiation (32). However, the fertility of *Gdpd5* KO mice has not been determined. We analyzed the other 2 Te-enriched family genes *Gdpd1* and *Gdpd4*. To analyze the physiological role of GDPD1 and GDPD4, we generated *Gdpd1* and *Gdpd4* mutant mice by CRISPR/Cas9 (via plasmid DNA microinjection) (33). Both mutant mice had no overt developmental or fertility abnormalities (average litter size: *Gdpd1* mutant males: 9.0 ± 1.9; *Gdpd4* mutant males: 9.0 ± 1.1; mean ± SD). Additional details are described in *SI Appendix*, Figs. S7 and S8.

### Localization of LYPD4 in Mouse Te and Spermatozoa.

**Fig. 5.**
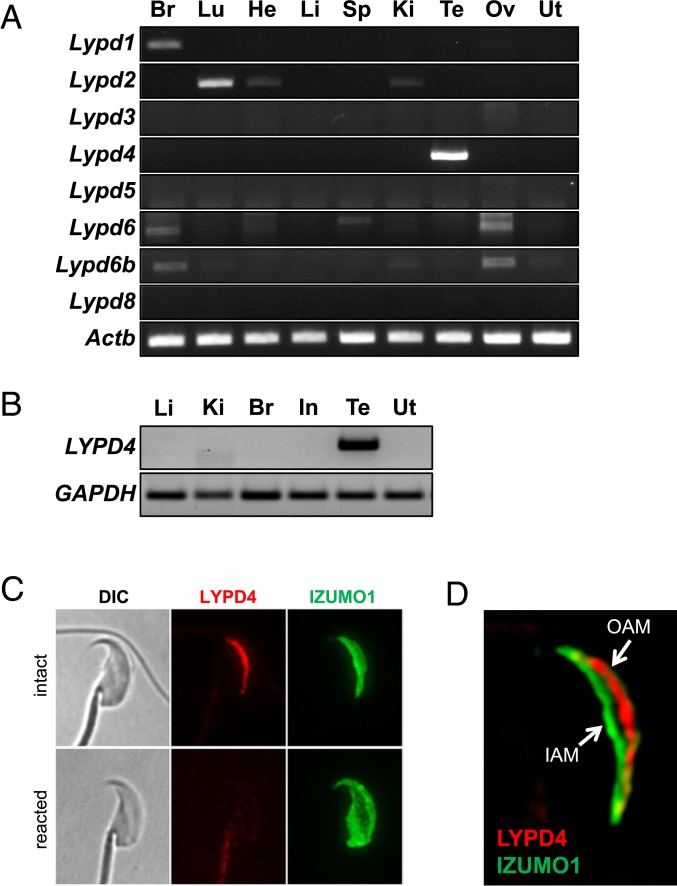
Characterization of LYPD4 in mice and humans. (*A*) Te-specific expression of mouse *Lypd4* by multitissue RT-PCR analysis. The expression of each gene was examined by RT-PCR using RNA isolated from various organs. *Lypd4* was detected only in the mouse Te. The *Actb* gene was used as an expression control. (*B*) RT-PCR analysis of human *LYPD4* in the human tissues. Human *LYPD4* was also detected only in the Te. (*C*) Immunostaining of LYPD4 in Cau Epi spermatozoa. LYPD4 (red signal) localized to the sperm acrosomal membrane. IZUMO1 (green signals) is a sperm acrosome membrane protein used as a marker for the acrosome reaction. (*D*) Confocal microsocopic observation of LYPD4 and IZUMO1 in Cau Epi spermatozoa. Although IZUMO1 (green signal) was detected on the outer acrosomal membrane (OAM), LYPD4 (red signal) localized to the inner acrosomal membrane (IAM).

In mice, there are 8 *Lypd* family genes (*Lypd1–6*, *6b*, and *8*). *Lypd2* and *Lypd4* showed tissue-specific expression by RT-PCR analysis while the other 6 genes had very weak bands in these organs (Fig. 5*A*). The expression of these genes was confirmed by *in silico* data (*SI Appendix*, Table S1). We found that *Lypd4* is exclusively expressed in the mouse and human Te (Fig. 5*B*), and the LYPD4 protein sequence is highly conserved in mammals (*SI Appendix*, Fig. S9*A*). To analyze the localization of LYPD4, we performed immunostaining of TGCs and Cau epididymal spermatozoa with SPACA1 as a marker for the inner acrosomal membrane and IZUMO1 as a marker for the acrosomal membrane. LYPD4 was localized to the outer acrosomal membrane in Te spermatozoa (*SI Appendix*, Fig. S9*B*). In Cau Epi spermatozoa, LYPD4 was not detected in acrosome-reacted spermatozoa (Fig. 5*C*). Moreover, LYPD4 and IZUMO1 showed different localizations of acrosomal membranes by confocal microscopy (Fig. 5*D*). These results suggest that LYPD4 is a sperm outer acrosomal membrane protein and disappears from the sperm head after the acrosome reaction.

### Male Fertility of *Lypd4* KO Mice and Fertilizing Ability of *Lypd4* KO Spermatozoa.

**Fig. 6.**
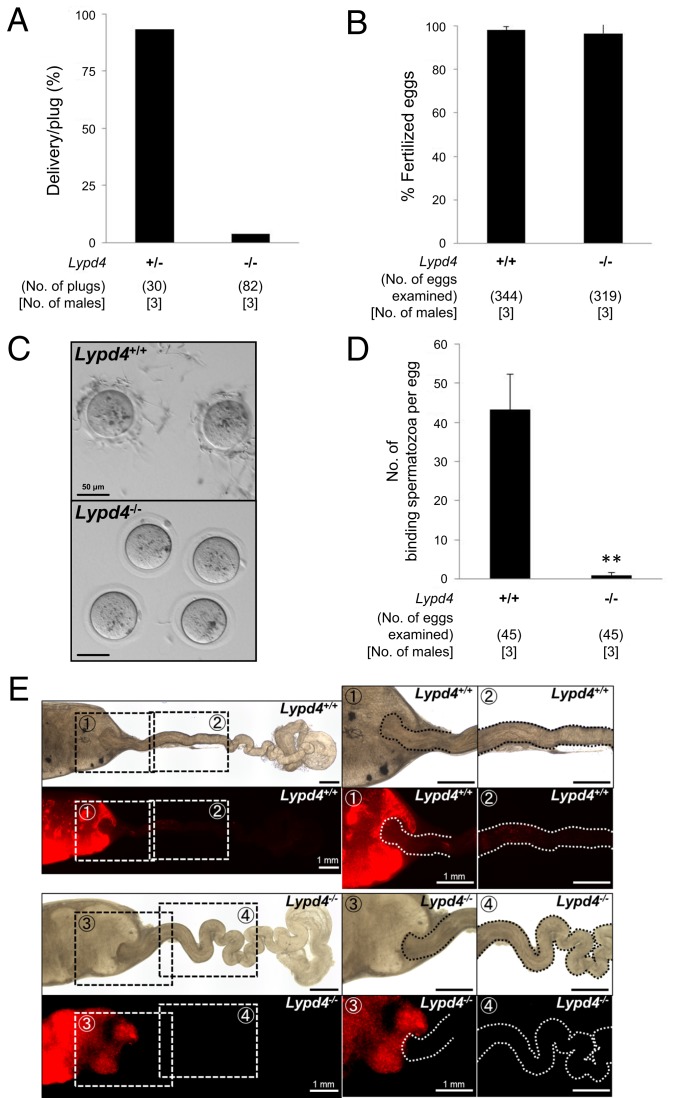
Male fertility and in vitro fertilizing ability in *Lypd4* KO mice. (*A*) Pregnancy rates of WT female mice mated with *Lypd4*^*+/−*^ and *Lypd4*^−/−^ male mice. Pregnancy rate is the success rate from natural matings (delivery per plug). The average pregnancy rate of females coupled with *Lypd4*^*+/−*^ and *Lypd4*^−/−^ male mice were 93.3% (28/30) and 3.7% (3/82), respectively. (*B*) In vitro fertilization rates using WT and *Lypd4* KO spermatozoa. Average fertilization rates of WT and *Lypd4* KO spermatozoa were 98.0 ± 1.5% (337/344 eggs) and 96.6 ± 7.0% (308/319 eggs), respectively. (*C*) Observation of ZP-binding in WT and *Lypd4* KO spermatozoa. *Lypd4* KO spermatozoa have an impaired ZP-binding ability in vitro (Scale bars: 50 μm.) (*D*) Average number of ZP-binding spermatozoa in vitro. The number of ZP-binding spermatozoa in *Lypd4* KO mice (1.0 ± 0.8 spermatozoa; mean ± SD) was significantly reduced compared with that of WT mice (43.2 ± 9.1 spermatozoa). **P < 0.01, Student’s *t* test. (*E*) Observation of female reproductive tract. Ut and oviducts from WT females mated with WT and *Lypd4* KO males carrying fluorescent protein-tagged spermatozoa indicated the failure of *Lypd4* KO spermatozoa to pass through the UTJ. Photographs were taken 4 h after coitus (Scale bars: 1 mm.)

To examine the function of LYPD4, we produced *Lypd4* KO mice by conventional ES cell methods (*SI Appendix*, Fig. S10*A*). *Lypd4* KO mice were confirmed by genotyping PCR and immunoblot analysis (*SI Appendix*, Fig. S10 *B* and *C*). Furthermore, *Lypd4* KO mice had no deleterious effects when Te histology and sperm morphology were examined (*SI Appendix*, Fig. S10 *D* and *E*). To determine male fertility, we checked the pregnancy rate in *Lypd4* KO mice. The pregnancy rate (delivery per plug) was 93.3% (28/30 plugs) of female mice mated with heterozygous KO (+/−) males (*n* = 3), but only 3.7% (3/82 plugs) of females mated with homozygous KO (−/−) males (*n* = 3) (Fig. 6*A*). These data indicate that *Lypd4* KO males are severely subfertile despite showing normal mating behavior with successful ejaculation and vaginal plug formation. To analyze the infertility of *Lypd4* KO males, we performed IVF assays using Cau Epi spermatozoa from *Lypd4* KO males mixed with cumulus-intact oocytes. *Lypd4* KO spermatozoa fertilize oocytes as effectively as WT spermatozoa (98.0 ± 1.5% [337/344 eggs] and 96.6 ± 7.0% [308/319 eggs] by WT and KO, respectively) (Fig. 6*B*). Next, we checked the sperm ZP-binding ability of the spermatozoa mixed with cumulus-free oocytes. *Lypd4* KO spermatozoa had a dramatically reduced ability to bind to the ZP compared with WT spermatozoa (43.2 ± 9.1 and 1.0 ± 0.8 spermatozoa per egg in the WT and KO spermatozoa, respectively; *P* < 0.01) (Fig. 6 *C* and *D*). To observe the behavior of ejaculated spermatozoa in vivo, we crossed the *Lypd4* KO mice with a transgenic mouse line in which the acrosome and mitochondria could be visualized by EGFP and DsRed2, respectively. When we observed the ejaculated spermatozoa through the wall of the female reproductive tract, we found almost equal amounts of spermatozoa in the Ut of WT females mated with WT males compared to *Lypd4* KO males. However, *Lypd4* KO spermatozoa were unable to migrate through the oviduct (Fig. 6*E*). These results indicate that *Lypd4* KO spermatozoa have impaired sperm migration through the oviduct in vivo and impaired ZP-binding ability in vitro.

### Immunoblot Analysis of TGCs and Spermatozoa in *Lypd4* and ADAM3-Associated KO Mice.

**Fig. 7.**
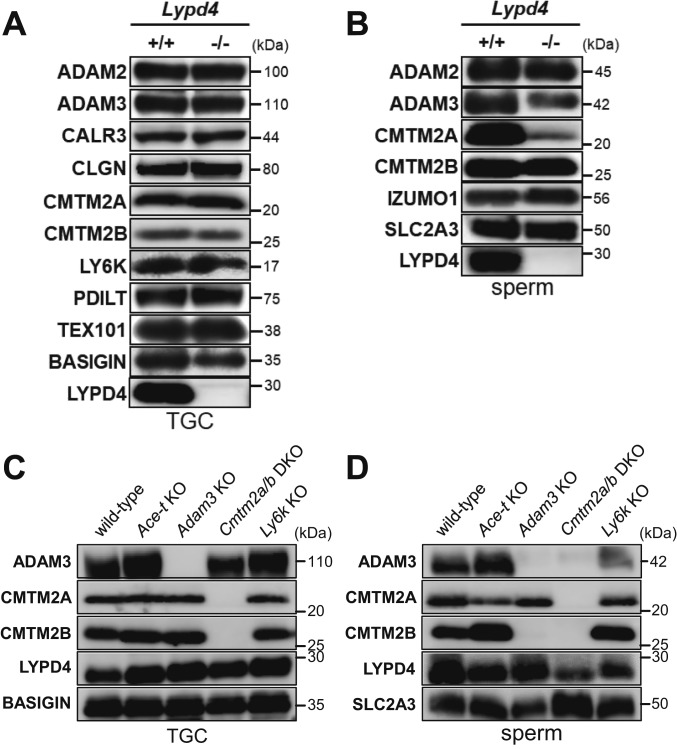
Immunoblot analysis of *Lypd4* KO and ADAM3-associated KO mouse lines. (*A*) Immunoblot analysis using TGC lysates collected from WT and *Lypd4* KO mice. There were no differences between WT and *Lypd4* KO mice. (*B*) Immunoblot analysis using sperm lysates collected from WT and *Lypd4* KO mice. Although ADAM3 and CMTM2A reduced in *Lypd4* KO mice, they remained. (*C* and *D*) Immunoblot analysis using TGC (*C*) and sperm (*D*) lysates collected from WT, *Ace-t*, *Adam3*, *Cmtm2a/b*, and *Ly6k* KO mice. LYPD4 remained in TGCs and spermatozoa from WT and these 4 genes’ KO mice. BASIGIN and SLC2A3 were used as loading controls.

The impaired sperm migration and the impaired sperm ZP-binding phenotypes in *Lypd4* KO mice are shared among mice lacking sperm membrane protein ADAM3 (27). Thus, we performed immunoblot analysis of ADAM3-associated proteins (membrane proteins: ADAM2, ADAM3, CMTM2A, CMTM2B, LY6K, and TEX101; ER chaperones: CALR3, CLGN, and PDILT) in *Lypd4* KO mice. There were no differences between WT and *Lypd4* KO TGCs (Fig. 7*A*). Notably, all of them (including ADAM3) remained in *Lypd4* KO spermatozoa, unlike the phenotype of previously reported ADAM3-associated KO mouse lines (Fig. 7*B*). ADAM3 was distributed in both the detergent-depleted and the detergent-enriched phases of Triton X-114 extracts from WT and *Lypd4* KO spermatozoa, although ADAM3 reduced in both phases of *Lypd4* KO spermatozoa compared to WT spermatozoa (*SI Appendix*, Fig. S10*F*). However, there is a possibility that ADAM3 and ADAM3-associated proteins (ACE, LY6K, and CMTM2A/B) interact with LYPD4 in mouse Te and spermatozoa because ADAM3 localizes aberrantly onto spermatozoa in *Ace* KO mice and disappears from spermatozoa in *Cmtm2a/b* double KO (DKO) mice (30, 34). CMTM2B is absent in *Adam3* KO spermatozoa, while CMTM2A remains (30). Moreover, KO spermatozoa from the TGC-specific GPI-anchored protein LY6K has the same phenotype in which the spermatozoa lose their fertilizing ability, but ADAM3 remains (25), which is similar to the results in *Lypd4* KO mice. Therefore, we checked LYPD4 levels in these four lines of KO mice (*Ace-t*, *Adam3*, *Cmtm2a/b*, and *Ly6k*) by immunoblot analysis after generating Te *Ace* (*Ace-t*) KO mice (*SI Appendix*, Fig. S11). LYPD4 shows no differences in expression among these KO TGCs and spermatozoa (Fig. 7 *C* and *D*). These results suggest that there is no direct interaction between ADAM3 and LYPD4 in spermatozoa and that LYPD4 is a new factor regulating sperm migration through the oviduct.

## Discussion

Mammalian fertilization is composed of many steps including sperm survival in the Ut, sperm migration in the female reproductive tract, physiological and morphological changes to the spermatozoa, and sperm egg interactions in the oviduct (27). Mammalian spermatozoa are morphologically differentiated in the Te, but Te spermatozoa are incapable of fertilization. Spermatozoa have to gain their fertilizing ability during spermiogenesis and Epi transit. We previously reported molecular mechanisms of spermatogenesis, sperm fertilizing ability, and Epi sperm maturation using genetically modified mice (25, 30, 31, 35–45). We also have established a quick and efficient method to make mutant mice using the CRISPR/Cas9 system (2, 6, 7, 33), allowing us to discover essential proteins for male fertility that are not only encoded by single genes, but also by gene clusters.

In this paper, we focused on Te- and Epi-enriched genes and generated genetically modified mice which have single gene disruptions (*Gdpd1*, *Gdpd4*, and *Lypd4*) and gene cluster dels (*Cst* and *Pate*). We found that the two gene clusters and *Lypd4* are required for sperm fertilizing ability in mice. Although the mutant mice produced normal-appearing spermatozoa, these Epi spermatozoa showed impaired sperm migration through the oviduct and impaired sperm-ZP binding in vitro. There is a strong connection between sperm migration through the UTJ and sperm-ZP binding ability as observed by time-lapse imaging of the female reproductive tract after natural mating (46); spermatozoa apparently attach around the opening of the UTJ and then migrate into the UTJ. Both phenotypes are shared with mutant mice that lack ADAM3. The sperm membrane protein ADAM3 is thought to play a pivotal role in sperm-ZP binding and sperm migration through the UTJ (28, 29). Although ADAM3 disappears from spermatozoa in *Cst* and *Pate* gene cluster del mice as expected, ADAM3 remains in *Lypd4* KO mice (Figs. 2*D*, 4*D*, and 7*B*). Thus, CST and PATE epididymal proteins regulate the stability of sperm ADAM3, but disruption of sperm acrosomal membrane protein LYPD4 does not affect ADAM3 localization in spermatozoa. A similar ADAM3-independent mechanism is observed in *Ly6k* and *Pgap1* KO mice (25, 26).

We generated CRISPR/Cas9-mediated mutant mice lacking the 2 genomic regions, *Cst* (114 kb between *Cstl1* and *Cstdc2*) and *Pate* (841 kb between *Pate8* and *Pate10*) gene clusters, and found that ADAM3 disappeared from these mutant spermatozoa. Eight *Cst* family genes are divided into 3 expression patterns; Te-enriched genes (*Cstl1*, *Cstdc1*, *Cst13*, and *Cst9*), Epi-enriched genes (*Cst11* and *Cstdc2*), and both Te- and Epi-enriched genes (*Cst8* and *Cst12*) (Fig. 3*B*). Also, in the *Pate* gene cluster, there are 12 noncoding genes, the functions of which have yet to be determined (Fig. 1*A*). Although we identified 2 genomic regions critical for male fertility, further investigations will be required to narrow down the region to reveal an essential gene, genes, and/or region for the sperm fertilizing ability. These large scale (more than 100-kb) reverse genetic approaches have been enabled by the emergence of the CRISPR/Cas9 system.

GDPDs are periplasmic and cytosolic proteins that are critical for the hydrolysis of deacylated glycerophospholipids to glycerol phosphate and alcohol, which are then utilized as a major source of carbon and phosphate (47). Since GDPDs are highly conserved from bacteria to mammals, there are 7 homologs (GDE1–7) in mice and humans. GDE2 (also known as GDPD5) is expressed in undifferentiated progenitors, but not in mature motor neurons, and the GPIase activity of GDE2 promotes this process during neural differentiation (48). Recently, there have been several reports of substrate-specific GPIase proteins, such as NOTUM, PGAP6 (officially named TMEM8), and angiotensin-converting enzyme (ACE) in mammals (48–51). To find a GDPD possessing the GPIase activity other than ACE (31), we analyzed the physiological roles of Te-enriched genes *Gdpd1* and *Gdpd4* using CRISPR/Cas9-mediated mutant mice. Unfortunately, we were unable to find an essential male GC-specific enzyme with GPIase activity since these mutant mice were fertile. Further experiments are required to discover the key gamete-specific GPIase protein. It is possible that these various GPIase enzymes play redundant roles and we will need to generate multi-KO mouse models to solve this riddle.

LYPD4 is a member of the LY6/PLAUR protein family (LYPD1-6, -6B, and -8) and is localized to the sperm acrosomal membrane. Most of the proteins in this family are GPI anchored, and as such, LYPD4 may be a GPI-AP that is localized to the sperm head. The other membrane protein, LYPD6, is enriched in synaptic loci, and *Lypd6* KO mice show reduced anxiety-like behavior and enhanced response to nicotine (52). LYPD8 is expressed in epithelial cells of the large intestinal gland and is essential to promote the segregation of flagellated microbiota and colonic epithelia (53). Physiological functions of other proteins in the LY6/PLAUR family remain to be determined in vivo. In this study, we found that ADAM3 remained in *Lypd4* KO mice (Fig. 7 *A* and *B* and *SI Appendix*, Fig. S10*F*) and LYPD4 did not disappear in *Ace*, *Adam3*, and *Cmtm2a/b* KO spermatozoa (Fig. 7*D*). Male infertility of *Lypd4* KO mice may be caused by abnormalities in sperm protein(s) other than ADAM3. Further experiments may be required to examine the cause of male infertility in *Lypd4* KO mice and how sperm acrosomal membrane protein LYPD4 is affected to gain sperm fertilizing ability. Moreover, we recently identified TGC-specific GPI-AP LY6K, which is essential for sperm migration through UTJ and ZP binding (25). We showed here that *Ly6k* KO spermatozoa retained not only ADAM3, but also LYPD4 (Fig. 7 *C* and *D*). These results suggest that LY6K and LYPD4 regulate sperm fertilizing ability via an as of yet undefined pathway in Te and spermatozoa. Therefore, our *Ly6k* and *Lypd4* KO mice may prove useful in elucidating the physiological functions of human LY6K and LYPD4. In this study, however, we could not clarify the localization of LYPD4 in *Pgap1* KO spermatozoa because most of the *Pgap1* KO mice showed postnatal lethality (26). Our findings support a potential role of human LYPD4 in sperm function and could be used to develop infertility treatments as well as male-specific contraceptives.

## Methods

### Animals.

All animal experiments were approved by the Animal Care and Use Committee of the Research Institute for Microbial Diseases, Osaka University. Human tissues were collected as nonhuman subject research by the Human Tissue Acquisition & Pathology (HTAP) Core at Baylor College of Medicine under the institutional review board (IRB) approved Protocol H-14435. Mice were maintained under a 12-h light/dark cycle (lights on from 8:00–20:00). WT mice were purchased from CLEA Japan (Tokyo, Japan) and Japan SLC (Shizuoka, Japan). In this study, we generated genetically modified mouse lines, (*Pate8*–*Pate10*)^*del*/*del*^ mice (Stock Del[*Gm17677*–*Gm17689*]1Osb); RBRC 09843 and Center for Animal Resources and Development (CARD) ID: 2463, *Gdpd1* mutant mice (B6D2-*Gdpd1* < em1Osb>); RBRC09986, *Gdpd4* mutant mice (B6D2-*Gdpd4* < em1Osb>); RBRC10115, *Lypd4* KO mice (STOCK-*Lypd4* < tm1Osb>/4E and STOCK-*Lypd4* < tm1Osb>/6A); RBRC05875 and RBRC05877, and *Ace-t* KO mice (STOCK-*Ace* < em1Osb>); RBRC09959. These were deposited to the RIKEN BioResource Research Center (https://mus.brc.riken.jp/en/) and the CARD, Kumamoto University (http://card.medic.kumamoto-u.ac.jp/card/english/). The *Adam3*, *Cmtm2a/b*, and *Ly6k* KO mouse lines were described previously (25, 30).

### RT-PCR Analysis.

Mouse cDNA was prepared from multiple adult tissues of WT mice (42). Briefly, using TRIzol reagent (Invitrogen, USA), total RNA was isolated from multiple adult tissues of WT mice and multiple adult human tissues obtained from the HTAP Core. Informed consent of these human tissues was obtained. Mouse and human cDNA were prepared using SuperScript III Reverse Transcriptase (Invitrogen, USA) following the manufacturer’s instruction. The amplification conditions were 1–3 min at 94 °C, followed by 30–40 cycles of 94 °C for 30 s, 55 or 65 °C for 30 s, and 72 °C for 30 s with a final 2–7 min extension at 72 °C. The primers used are listed in *SI Appendix*, Tables S2 and S3.

### Generation of (*Pate1*–*Pate3*)^*del/del*^, (*Pate8*–*Pate10*)^*del/del*^, and (*Cstl1-Cstdc2*)^*del/del*^ Mice with CRISPR/Cas9.

(*Pate1*–*Pate3*)^*del/del*^ mice were produced by introducing gRNAs and the CAS9 enzyme (Thermo Fisher Scientific) into fertilized eggs with an electroporator (EP) (NEPA21, Nepagene) (3). *(Pate10-Pate8)*^*del/del*^ mice and (*Cstl1-Cstdc2*)^*del/del*^ mice were produced by injection of pX459 plasmid (https://www.addgene.org/62988/) into mouse ES cells as described previously (6, 7). A search for sgRNA and off-target sequences was performed using Benchling (https://benchling.com) (54) or CRISPRdirect software (https://crispr.dbcls.jp/) (55). The sgRNA sequences used for the EP were: 5′-CATTGGAGTTCAATGTAATG-3′ for the 11th exon of *Pate2* and 5′-TGTCCTTGATGCTTACAGGG-3′ for the third exon of *Pate3*. The sgRNA sequences used for transfection were: 5′-AGGTATTTTCCAATTGCAGT-3′ for the first exon of *Pate8* and 5′-TTGTACAAGTCTCATTCAAT-3′ for the first exon of *Pate10*. Additionally, the sgRNA sequences used for transfection were: 5′-CACAGCAGTCTTGCCGGCAT-3′ for the second exon of *Cstl1*, and 5′-AGATTGTCCCATGTAACACT-3′ for the first exon of *Cstdc2.* The mutant ES cells were injected into 8-cell stage embryos, and then they were transferred into the Ut of pseudopregnant ICR females the next day. Screening of the obtained mutant mice was performed by direct sequencing following PCR. The primers used are listed in *SI Appendix*, Table S2 and S3. Detailed genotype information of mutant mouse lines was shown in Figs. 1*E* and 3*E* and *SI Appendix*, Fig. S1*D*.

### Male Fertility Test.

Sexually mature mutant male mice were caged with 2-mo-old B6D2F1 or mutant females for several months, and the number of pups in each cage was counted within a week of birth. Pregnancy rates are presented as the success rates for getting pregnant from natural matings. Average litter sizes are presented as the number of total pups born divided by the number of litters for each genotype.

### In Vitro Fertilization.

In vitro fertilization using mouse spermatozoa was performed as described previously (39).

### Sperm ZP-Binding Assay.

Sperm ZP-binding assay was performed as described previously (34). Briefly, 30 min after mixing with 2-h-incubated spermatozoa, cumulus-free eggs were fixed with 0.25% glutaraldehyde. The bound spermatozoa were observed with an Olympus IX73 microscope.

### Statistical Analysis.

Statistical analyses were performed using Student’s *t* test and Mann–Whitney *U* test inserted into Microsoft Excel after the data were tested for normality of distribution. Differences were considered significant at *; *P* < 0.05 and **; *P* < 0.01.

## Supplementary Material

Supplementary File
