# Supplementary material for: Identification of multiple male reproductive tract-specific proteins that regulate sperm migration through the oviduct in mice
Source: Proc Natl Acad Sci U S A. 2019 Aug 27;116(37):18498–506. doi: 10.1073/pnas.1908736116 (PMC6744855; doi:10.1073/pnas.1908736116)
Supplement: Supplementary File [file pnas.1908736116.sapp.pdf]

PNAS
www.pnas.org

Supplementary Information for

Identification of multiple male reproductive tract-specific proteins that regulate sperm migration through the oviduct in mice

Yoshitaka Fujihara[1,2,3,4#], Taichi Noda[1,2#], Kiyonori Kobayashi[1,5#], Asami Oji[1,2], Sumire Kobayashi[1,2], Takafumi Matsumura[1,2], Tamara Larasati[1,6], Seiya Oura[1,2], Kanako Kojima-Kita[1,6], Zhifeng Yu[3], Martin M. Matzuk[3]*, and Masahito Ikawa[1,2,6,7]*

[1]Research Institute for Microbial Diseases, Osaka University, 3-1 Yamadaoka, Suita, Osaka 565-0871, Japan. [2]Graduate School of Pharmaceutical Sciences, Osaka University, 1-6 Yamadaoka, Suita, Osaka 565-0871, Japan. [3]Center for Drug Discovery and Department of Pathology & Immunology, Baylor College of Medicine, One Baylor Plaza, Houston, TX 77030. [4]Department of Bioscience and Genetics, National Cerebral and Cardiovascular Center, 6-1 Kishibeshinmachi, Suita, Osaka 564-8565, Japan. [5]Graduate School of Frontier Biosciences, Osaka University, 1-3 Yamadaoka, Suita, Osaka 565-0871, Japan. [6]Graduate School of Medicine, Osaka University, 2-2 Yamadaoka, Suita, Osaka 565-0871, Japan. [7]The Institute of Medical Science, The University of Tokyo, 4-6-1 Shirokanedai, Minato-ku, Tokyo 108-8639, Japan.

[#] Y.F., T.N., and K.K. contributed equally to this work.

* Corresponding authors:
Martin M. Matzuk, MD, PhD and Masahito Ikawa, PhD
Email: mmatzuk@bcm.edu and ikawa@biken.osaka-u.ac.jp

This PDF file includes:

Supplementary text
Figures S1 to S11
Tables S1 to S3
SI References

www.pnas.org/cgi/doi/10.1073/pnas.1908736116

Supplementary Information Text

RNA-seq RPKM values of genes expressed in multiple tissues. RNA-seq data are analyzed by the mouse ENCODE Consortium (A Comparative Encyclopedia of DNA Elements in the Mouse Genome) and are publicly available (1). C57BL/6 mice were used for all tissue resections. The following tissues were taken from 8-week old littermate: frontal lobe, genital fat pad that includes epididymis, heart, kidney, large intestine, liver, lung, ovary, placenta (from pregnant mice), small intestine, spleen, stomach, testis, and thymus. Genital fat pad and testis are indicated by blue and green, respectively. The values of *Pate8* and *Gm27235* were not shown in the data (indicated by gray).

Alignment of amino acid sequences for PATE family proteins. Amino acid sequences for PATE family proteins (accession #: CCDS57669 for PATE1, CCDS52756 for PATE2, CCDS52755 for PATE3, CCDS22967 for PATE4, CCDS52760 for PATE5, CCDS40577 for PATE6, CCDS52759 for PATE7, CCDS52767 for PATE8, CCDS52766 for PATE9, CCDS52758 for PATE10, CCDS52765 for PATE11, CCDS52764 for PATE12, ENSMUST00000176153 for PATE13, CCDS22970 for PATE14, ENSMUST00000184431 for GM27235, and CCDS52762 for GM5916) were alignment analysis with Clustal Omega (https://www.ebi.ac.uk/Tools/msa/clustalo/). The existence of signal peptide was examined with SignalP (http://www.cbs.dtu.dk/services/SignalP/).

Testis and epididymis histology and sperm morphology. After breeding studies, males were killed by cervical dislocation following anesthesia. Testes were weighed individually. Testes were fixed in 4% paraformaldehyde in PBS and were processed for paraffin embedding. Paraffin sections were cut 5 μm and stained with periodic acid-Schiff (PAS) and then counterstained with Mayer hematoxylin solution (Wako). The epididymal histology was observed by described previously (2). The cauda epididymal spermatozoa were dispersed in PBS and then the sperm morphology was observed under a phase-contrast microscope (BX50, Olympus).

Sperm motility analysis. Cauda epididymal spermatozoa were dispersed in TYH drops for sperm motility. After an incubation period of 10 and 120 minutes, the sperm motility pattern was examined using the CEROS sperm analysis system (software version 12.3; HamiltonThorne Biosciences) (3) and the default program (Mouse CytoD 4x dark field settings) of the CEROS II sperm analysis system (software version 1.5.2; Hamilton Thorne Biosciences).

Antibodies. The monoclonal antibodies used here were as described previously: TES101 for TEX101, 1D5 for ACE, KS64-10 for SLC2A3, and KS64-125 for IZUMO1 (4). Other antibodies were purchased from Thermo Fisher Scientific (PA5-23848 for LYPD4), Acris Antibodies (BP5112 for SPACA1), Santa Cruz Biotechnology (sc-365288 for ADAM3 and sc-9757 for BASIGIN), Cell signaling (2118 for GAPDH), and Chemicon (7C1 for ADAM3 and 9D for ADAM2). Rabbit antisera against CALR3, CLGN, CMTM2A, CMTM2B, LY6K, PDILT, SPACA1, and SPESP1 were as described previously (5-9). Dilutions used were 1:200 to 1:300 for immunostaining and 1:500 to 1:1000 for immunoblot analysis.

Immunoblot. Immunoblot analysis was performed as described previously (10). Briefly, testicular germ cells were collected from the seminiferous tubules of testes. Sperm samples were collected from caput, corpus, and cauda epididymis, respectively. These samples were homogenized in lysis buffer containing 1% Triton X-100 and 1% protease inhibitor (Nacalai Tesque, Kyoto, Japan) and then were centrifuged (10,000g for 20 min at 4℃), and the supernatants were collected. Protein lysates were separated by SDS/PAGE under reducing condition and transferred to PVDF membranes (Merck Millipore). After blocking, blots were incubated with primary antibodies overnight at 4℃, and then incubated with secondary antibodies conjugated with horseradish-peroxidase. The detection was performed using an ECL plus western blotting detection kit (GE Healthcare) and Chemi-Lumi One Ultra (Nacalai Tesque). For experiments of *Cst* and *Pate* family genes, we used simple western systems (Proteinsimple) with primary antibodies for ADAM2, ADAM3, and IZUMO1, and secondary antibodies [anti-rabbit secondary antibody (Proteinsimple,

cat# 042-206), anti-mouse secondary antibody (Proteinsimple, cat# 042-205), and horseradish peroxidase-conjugated goat anti-mouse IgG antibody (Jackson ImmunoResearch Laboratories)].

Generation of *Gdpd1* and *Gdpd4* mutant mice with CRISPR/Cas9. *Gdpd1* and *Gdpd4* mutant mice were produced by microinjection of pX330 plasmid (https://www.addgene.org/42230/) into mouse embryos as described previously (11, 12). A search for sgRNA and off-target sequences was performed using CRISPRdirect software (https://crispr.dbcls.jp/) (13). The sgRNA sequence used for microinjection were: 5'-GAGAAGGCAAAAAGCCGCGG-3' for the first exon of *Gdpd1* and 5'-CAGGGAGAATAAATCCCCAG-3' for the ninth exon of *Gdpd4* (targeted for the common exon of several variants). Each sgRNA was injected into the pronuclei of fertilized eggs. The 2-cell stage embryos were transferred into the oviducts of pseudopregnant ICR females the next day. *Gdpd1* mutant mice had a 20 bp deletion (5'-TCGTCCACCGCGGCTTTTTG-3') in the first exon. *Gdpd4* mutant mice had a 1 bp deletion (5'-G-3') and a 19 bp deletion (5'-ACTGGGGATTTATTCTCCC-3') in the ninth exon. The primers used are listed in Table S3. Detailed genotype information of mutant mouse lines is shown in Figure S7 and S8.

Immunostaining. Immunostaining was performed as described previously (8, 14). A confocal microscopic observation was performed as described previously (15).

Generation of *Lypd4* knockout mice. A 2.7-kb *Asc*I-*Kpn*I fragment as a short arm and a 5.6-kb *Not*I-*Sal*I fragment as a long arm were obtained by PCR using genomic DNA derived from C57BL/6N mice as a template. Both arms were inserted into a pNT1.1 vector (https://www.ncbi.nlm.nih.gov/nuccore/JN935771). After linearization with *Not*I digestion, the targeting vector was electroporated into EGR-G01 (129S2 x [CAG/Acr-Egfp]C57BL/6NCr) embryonic stem (ES) cells (16), and colonies were screened. To disrupt the *Lypd4* gene, fourth and fifth exons were replaced with a FRT-flanked neo cassette, and a thymidine kinase (tk) expression cassette was used for negative selection. After G418 selection, 14 of 96 drug-resistant clones had a homologous recombination event after PCR analysis. The mutant ES cell clones were injected into 8-cell stage ICR embryos, and the chimeric blastocysts were transferred into the uterine horns of pseudopregnant ICR females the next day. The obtained chimeric males were mated with B6D2F1 females for germ-line transmission. Offsprings from heterozygous intercrosses were genotyped by PCR. Both a 496-bp band as the wild-type allele and a 295-bp band as the knockout (KO) allele were amplified by PCR. The primers used are listed in Table S3.

Phase separation of Triton X-114 extracts of spermatozoa. Phase separation of Triton X-114 sperm extracts was performed as previously described with minor modification (17). Briefly, spermatozoa were collected from cauda epididymis and vas deferens into PBS and then centrifuged at 3,000 rpm for 5 min at 4℃. The sperm pellets were sonicated with a sonicator (SLPe, Branson Ultrasonics, CT, USA) in PBS that contained 1% Triton X-114 and 1% protease inhibitor cocktail. Sonicated samples were incubated for 1 h on ice with occasional vortexing. After centrifuging at 15,000 rpm for 30 min at 4℃, the supernatants were collected in new microtubes. After incubation at 37℃ for 15 min, the tubes were centrifuged at 2,500 rpm for 15 min at room temperature to separate the Triton X-114 extract into the detergent-depleted phase and detergent-enriched phase. The supernatants were collected as a detergent-depleted phase. After removing the inner layer, PBS was added to the pellet and re-suspended as a detergent-enriched phase. The separated detergent-depleted and detergent-enriched phases were mixed with SDS-sample buffer, and subjected to SDS-PAGE and immunoblot analysis.

Generation of *Ace-t* mutant mice with CRISPR/Cas9. Testicular *Ace* (*Ace-t*) transcribes from the thirteenth exon of *Ace* gene as the first coding exon. The thirteenth exon transcribes only for *Ace-t* expression. To disrupt only ACE-t, we designed sgRNAs into the thirteenth exon. *Ace-t* mutant mice were produced by the transfection of pX330 plasmids into mouse ES cells, EGR-G101 [C57BL/6N-Tg(*CAG/Acr-Egfp*) x C57BL/6N-Tg(*CAG/Acr-Egfp*)], as described previously (16, 18). A search for sgRNA and off-target sequence was performed using CRISPRdirect software (http://crispr.dbcls.jp/). After the validation of EGFP expression assay (12), the sgRNA sequence used for transfection were: 5'-GGCCAAGGTTGGGCTACTCC-3' and 5'-

TCTTTCTGCTGCTCTGCTGT-3’. Screening of ES cell clones was performed by direct sequencing following PCR. The primers used are listed in Table S3. 12 of 13 clones were introduced indel (insertion/deletion) mutations screened by direct sequencing. The mutant ES cell clones were injected into 8-cell stage ICR embryos, and chimeric blastocysts were transferred into the uterine horns of pseudopregnant ICR females the next day. The obtained chimeric males were mated with B6D2F1 females for germ-line transmission. *Ace-t* mutant mice had a 37 bp deletion (5’-CTCCAGGACTGCCCAGCTTCCTCTTTCTGCTGCTCTG-3’) in the thirteenth exon. The reproductive phenotype of *Ace* KO mice was reported previously (17).

A

MGI	Human ortholog	Signal peptide	C-C-C-C-C	C-C-CC-CN	Total no. of amino acids
Pate2	*PATE2*	YES	(28)..CYKCKKYHL-GLCYGIMTSCVPN----HRQTC..(22)	..CMTNCEDIN-FLSFERRTELICCKHSNYCN..(5)	111
Pate4	*PATE4*	YES	(25)..CNSCEKSRD-SRCTMSQSRCVAK----PGESC..(17)	..CSPQCKEKQ-LNTGKKLIYIMCCE-KNLCN..(2)	99
Pate3	*PATE3*	YES	(22)..CVTCHLRTQSDHCRRGFGVCLAQ----KHEIC..(17)	..CQRFCKNLT-YIFNNRTYTHKCCN-SDFCN..(3)	98
Pate1	*PATE1*	YES	(46)..CRMCHLKFPGENCTRGRGICTAT----AEEAC..(18)	..CLKNCANVKKIKWGPYLVDFRCCRGQDMCN..(3)	125
Pate10	NO	YES	(26)..CTSCDEYID-KTCTRNLGVCHAR---YPDFAC..(22)	..CPRRCVEYVRFIRFEKN-IFSCCN-ESYCN..(12)	116
Pate7	NO	YES	(22)..CRECKSYSH-QKCIHEMRTCTAK----DGESC..(21)	..CQENCTTDE-YYYGDYTILIKCCKKFDFCN..(10)	109
Gm27235	NO	YES	(21)..CVQCQSYKN-GECATKKETCTTK----PGETC..(21)	..IRHRRKIW--KIA-RLS	83
Pate6	NO	YES	(21)..CVQCQSYKN-GECATKKETCTTK----PGETC..(20)	..CTNSCKFEE-KTSGYLTTHTYCCSHGDFCN..(9)	106
Pate5	NO	YES	(27)..CMVCKSFKS-GHCLVGKNNCTTR----YKPGC..(22)	..CDKACMAEN-MYLGALKISTFCCKGEDFCN..(12)	117
Pate13	NO	YES	(29)..CNLCSHYDG-FKCRNGMKSCWKFDLWTQNRTC..(23)	..CK-PCAPGMYQMFHDLLRETFCCIDRNYCN..(25)	137
Gm5916	NO	YES	(27)..CMVCNSFKS-GHCSAGKNNCTTR----YKPGC..(22)	..CDKACMAES-RSHVSWSLEDIYLLLQR	102
Pate12	NO	YES	(23)..CITCDRINSQGICESGEGCCQAK----PGEKC..(17)	..CANICFTGT-VQTGDQTVKMKCCKKRSFCN..(2)	99
Pate11	NO	YES	(23)..CITCDRMNSRRICEGKEGCCQAR----PGEKC..(17)	..CADLCFTGT-VMIGDKTVKMNCCNNKSFCN..(2)	99
Pate9	NO	YES	(25)..CFQCTLFNSKGKCLFQEPPCETQ----NNEVC..(17)	..CSHTCVNQT-LNLRNKRIEMKCCNDKSFCN..(2)	101
Pate8	NO	YES	(33)..CVKCKHFNSTKNCLTQSGYCVAR----RDQKC..(17)	..CWTHCVNKY-IIRGSVRSEHKCCNSSSLCN..(2)	109
Pate14	NO	YES	(23)..CVSCGRLNSSGICETAETSCEAT----NNRKC..(17)	..CLGTCFNYT-KTNNNMVKEHKCCDHQNLCN..(2)	99

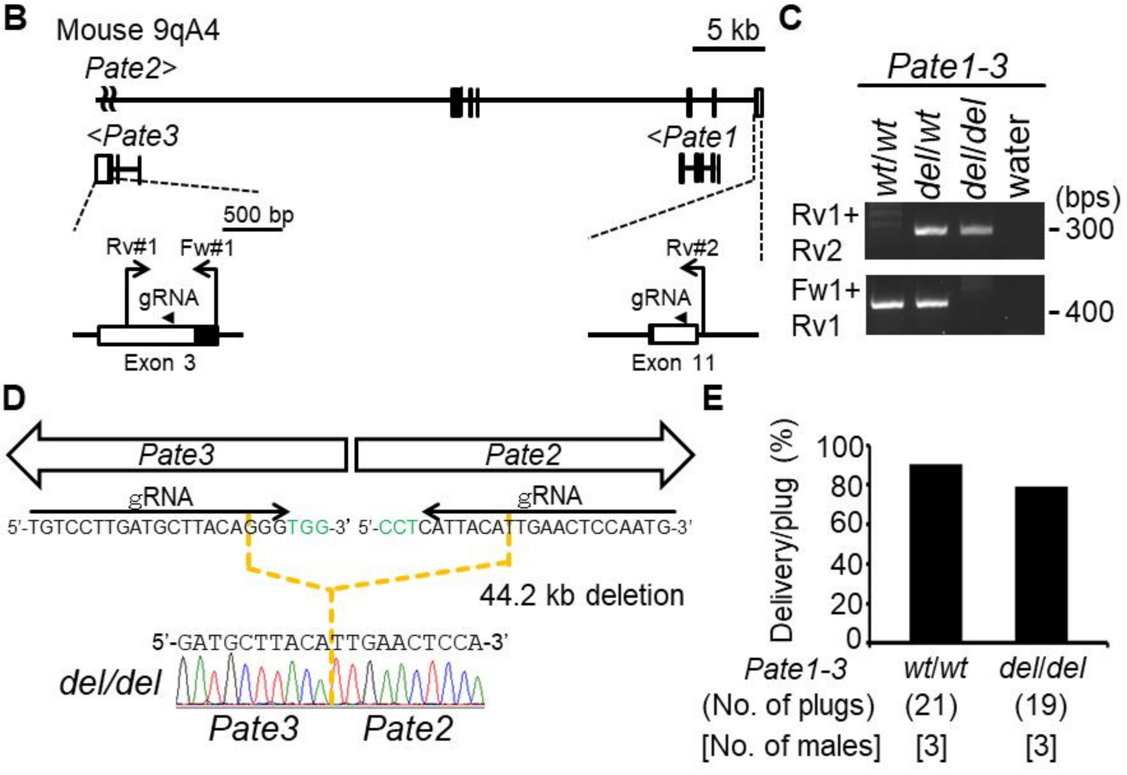

Fig. S1. Fecundity of (*Pate1-3*)$^{del/del}$ males. (A) Alignment of amino acid sequences of PATE family. The alignment of amino acid sequences of coding genes in the region between *Pate1* and *Pate14* was performed with Clustal Omega. The existence of human orthologs and the total number of amino acids were searched with MGI database. The signal peptide was detected by SignalP. All PATE family proteins conserve cysteine rich regions. (B) Production of (*Pate1-3*)$^{del/del}$ males. Black-colored regions in each exon and inequalities in each gene show the coding regions and the direction of transcription. Fw: forward primer, Rv: reverse primer. (C) Genotyping. Three primers (Fw#1, Rv#1, and Rv#2 in panel A) were used for PCR. (D) Sequencing of mutant allele. The 44.2 kb-region between *Pate1* and *Pate3* was deleted in (*Pate1-3*)$^{del/del}$ mice. (E) Fecundity of (*Pate1-3*)$^{del/del}$ males. The pregnancy rates of females mated with wild-type and (*Pate1-3*)$^{del/del}$ males were 90.5% (19/21) and 78.9% (15/19), respectively.

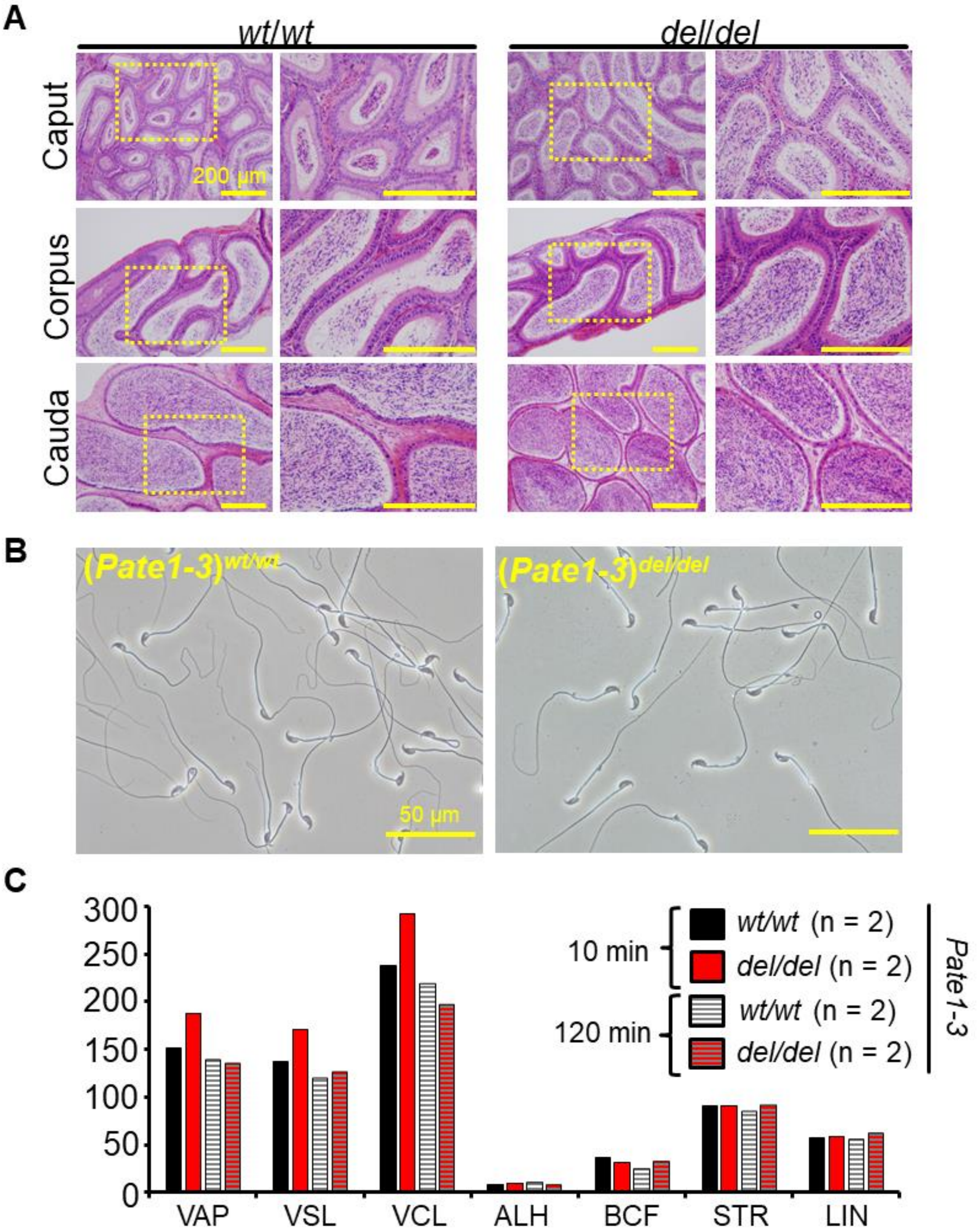

Fig. S2. Characteristics of epididymides from (*Pate1-3*)$^{del/del}$ males. (A) Observation of each region of an epididymis. There was no obvious defect in epididymal histology using H&E staining. (B) Sperm morphology. The morphology of (*Pate1-3*)$^{del/del}$ spermatozoa was comparable to the control. (C) Sperm motility. There was no obvious defect of motility parameters between wild-type and (*Pate1-3*)$^{del/del}$ spermatozoa. VAP: average path velocity, VSL: straight line velocity, VCL: curvilinear velocity, ALH: amplitude of lateral head, BCF: beat cross frequency, STR: straightness of trajectory, LIN: linearity.

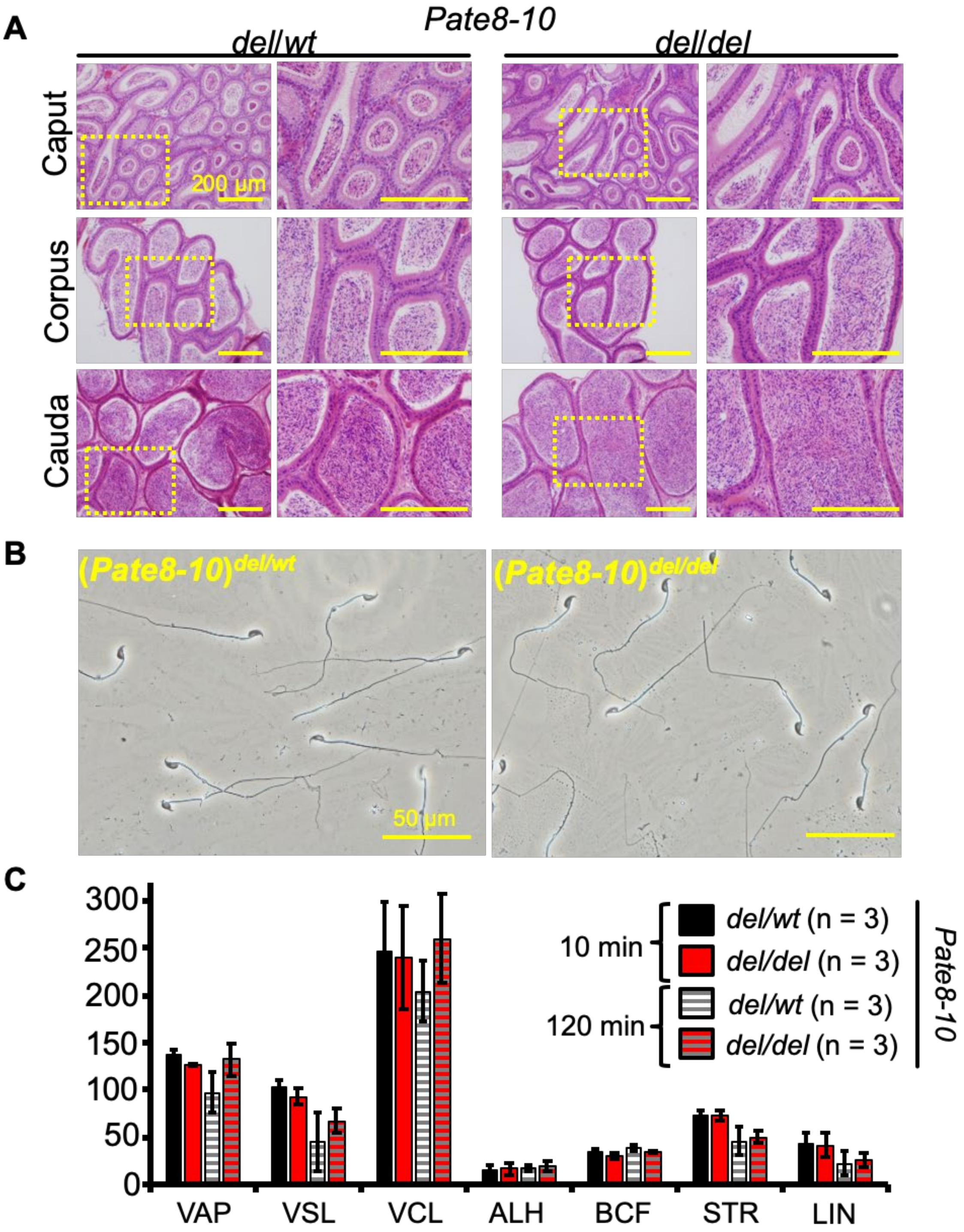

Fig. S3. Characteristics of epididymides from (*Pate8-10*)$^{del/del}$ males. (A) Observation of each region of an epididymis. There was no obvious defect in epididymal histology using H&E staining. (B) Sperm morphology. The morphology of (*Pate8-10*)$^{del/del}$ spermatozoa was comparable to the control. (C) Sperm motility. There was no difference in sperm motility parameters between (*Pate8-10*)$^{del/wt}$ and (*Pate8-10*)$^{del/del}$ spermatozoa. VAP: average path velocity, VSL: straight line velocity, VCL: curvilinear velocity, ALH: amplitude of lateral head, BCF: beat cross frequency, STR: straightness of trajectory, LIN: linearity.

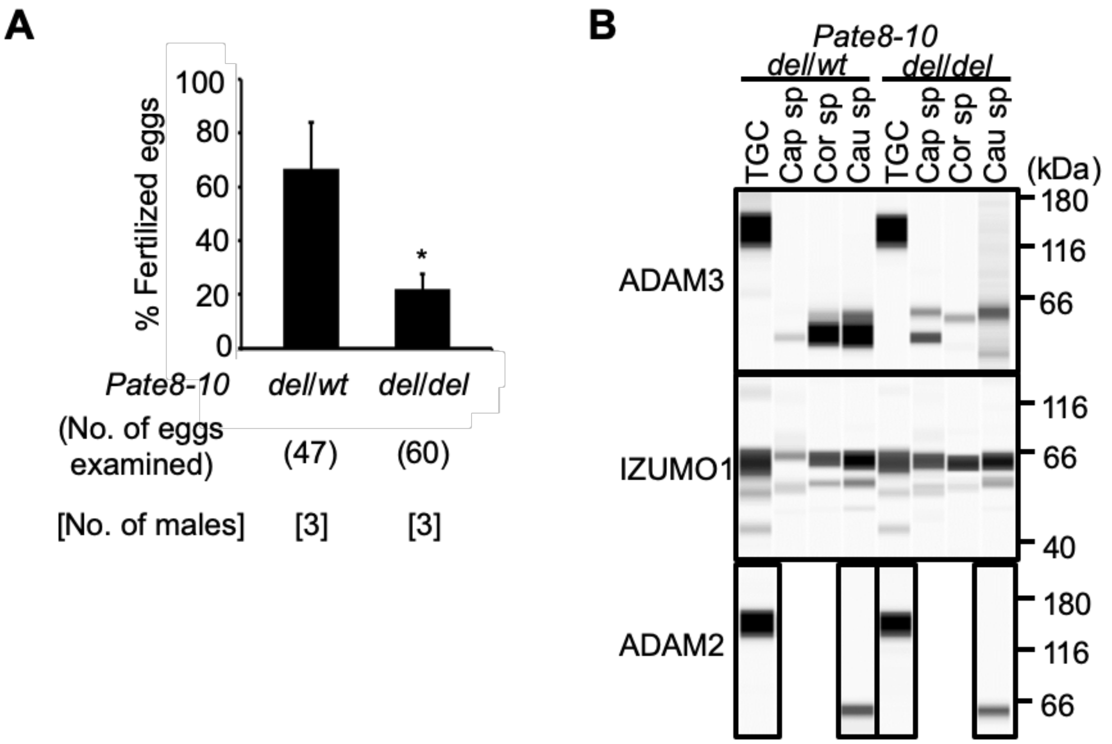

Fig. S4. Characterization of (*Pate8-Pate10*)$^{del/del}$ spermatozoa. (A) Sperm fertilizing ability using cumulus free oocytes *in vitro*. The fertilization rate of (*Pate8-10*)$^{del/del}$ spermatozoa significantly reduced due to the decrease of binding spermatozoa to zona pellucida (Fertilization rates, *del/wt*: 65.5 ± 17.8%, *del/del*: 19.5 ± 4.9%) (also see Figure 2C). *P < 0.05. (B) Detection of ADAM3 in TGC and each epididymis region. ADAM3 could not detect in the corpus epididymal spermatozoa from (*Pate8-10*)$^{del/del}$ mice. ADAM2 and IZUMO1 is used as the control. TGC: testicular germ cells, Cap sp: caput epididymal spermatozoa, Cor sp: corpus epididymal spermatozoa, Cau sp: cauda epididymal spermatozoa.

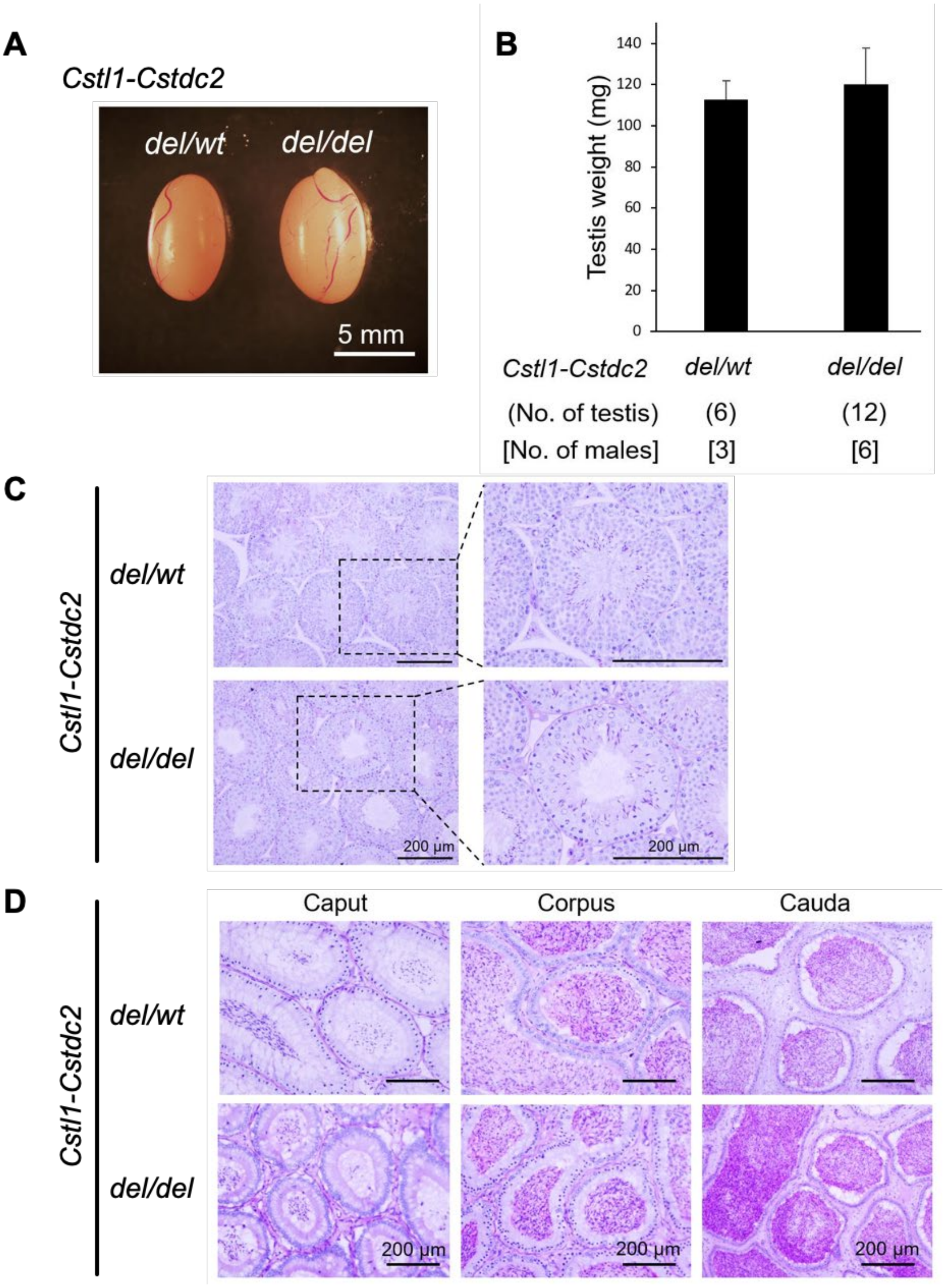

Fig. S5. Testicular and epididymal histology of (*Cstl1-Cstdc2*)$^{del/del}$ mice. (A) Observation of testicular morphology. Scale bars: 5 mm. (B) Testicular weights in (*Cstl1-Cstdc2*) mutant mice. Average testicular weights of (*Cstl1-Cstdc2*)$^{del/wt}$ and (*Cstl1-Cstdc2*)$^{del/del}$ mice were 112.7 ± 9.1 mg (n = 3) and 120.0 ± 17.9 mg (n = 6), respectively. (C) PAS staining of testicular sections. Right figures are magnified images of the boxes indicated in the left figures. Scale bars: 200 µm. (D) PAS staining of epididymal sections. Epididymides from (*Cstl1-Cstdc2*)$^{del/wt}$ and (*Cstl1-Cstdc2*)$^{del/del}$ mice indicated in the upper and lower figures, respectively. Scale bars: 200 µm.

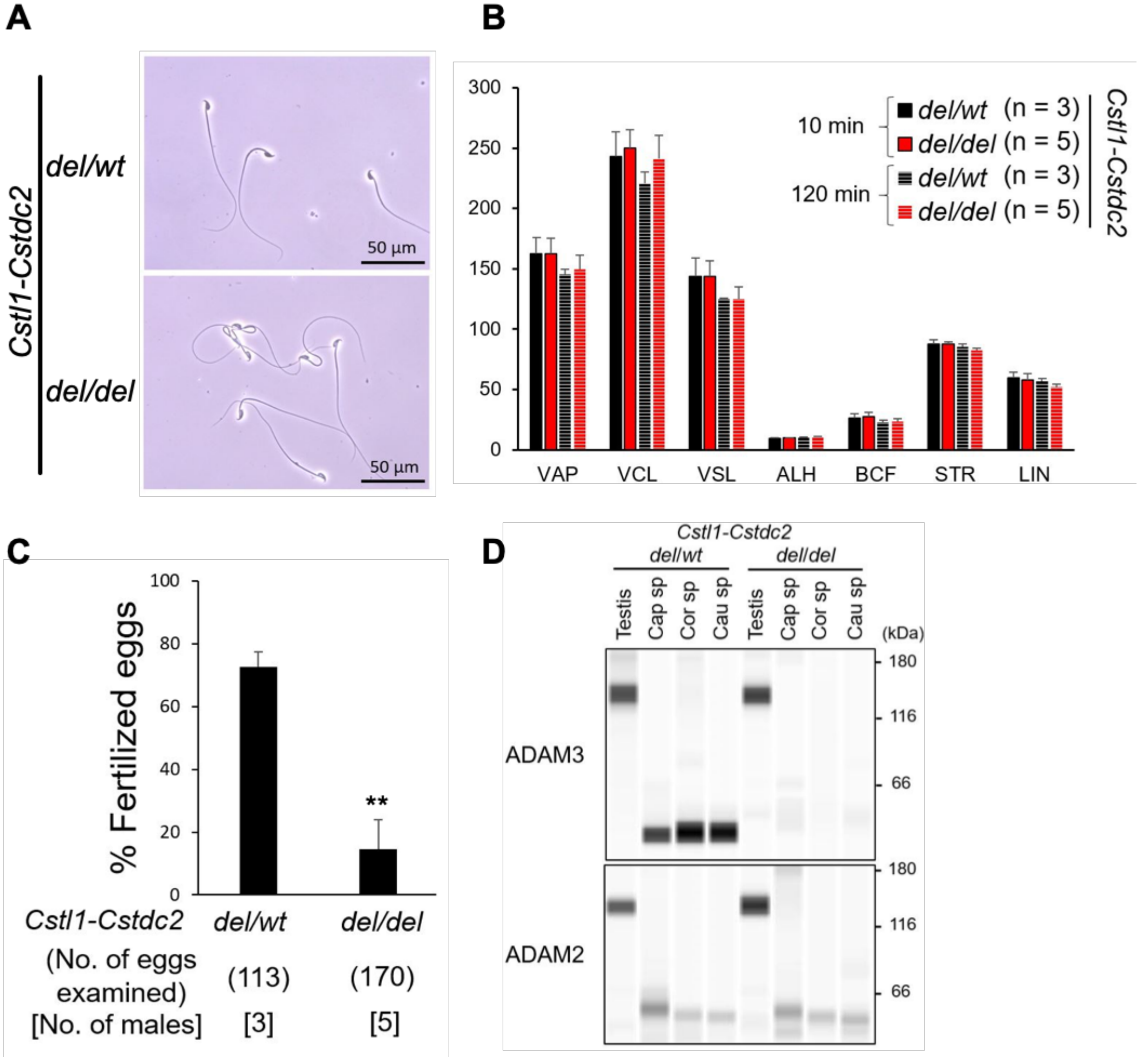

Fig. S6. Characterization of (*Cstl1-Cstdc2*)$^{del/del}$ spermatozoa. (A) Cauda epididymal spermatozoa in (*Cstl1-Cstdc2*) mutant mice. Scale bars: 50 μm. (B) Sperm motility. There was no difference in sperm motility parameters between (*Cstl1-Cstdc2*)$^{del/wt}$ and (*Cstl1-Cstdc2*)$^{del/del}$ spermatozoa. VAP: average path velocity, VSL: straight line velocity, VCL: curvilinear velocity, ALH: amplitude of lateral head, BCF: beat cross frequency, STR: straightness of trajectory, LIN: linearity. (C) *In vitro* fertilizing ability of (*Cstl1-Cstdc2*) mutant spermatozoa using cumulus-free oocytes. Average of fertilization rates of (*Cstl1-Cstdc2*)$^{del/wt}$ and (*Cstl1-Cstdc2*)$^{del/del}$ spermatozoa were 72.7 ± 4.8 % and 14.6 ± 9.3 %, respectively. **P < 0.01, Student's t-test. (D) Immunoblot analysis of ADAM3 in testis and epididymis. ADAM3 could not detect in the caput epididymal spermatozoa from (*Cstl1-Cstdc2*)$^{del/del}$ mice. ADAM2 is used as the control. Cap sp: caput epididymal spermatozoa, Cor sp: corpus epididymal spermatozoa, Cau sp: cauda epididymal spermatozoa.

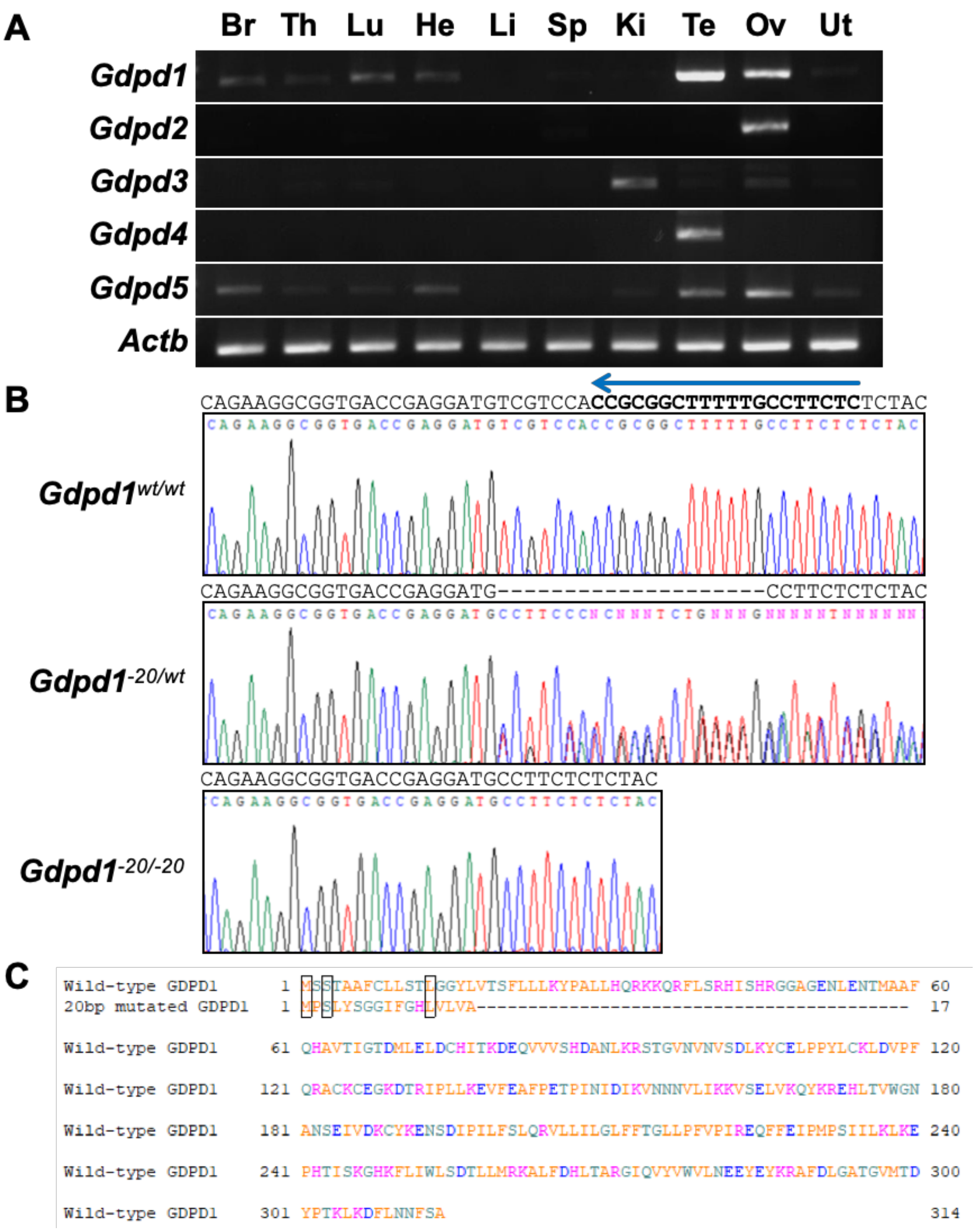

Fig. S7. Characterization of *Gdpd* family genes in mice and generation of *Gdpd1* mutant mice with CRISPR/Cas9. (A) Multi-tissue RT-PCR analysis of *Gdpd* family genes in mice. *Gdpd1*, *Gdpd4*, and *Gdpd5* were expressed strongly in the testis. The *Actb* gene was used as an expression control. Br: brain, Th: thymus, Lu: lung, He: heart, Li: liver, Sp: spleen, Ki: kidney, Te: testis, Ov: ovary, Ut: uterus. (B) Waveforms of direct sequencing of the 20 bp deletion (5'-TCGTCCACCGCGGCTTTTTG-3') in *Gdpd1* gene. Arrow indicates gRNA sequence. (C) Amino acid sequence of *Gdpd1* mutant mice. The 20 bp deletion caused a frameshift mutation leading to a premature termination codon after the amino acid 17th (314 amino acids in wild-type mice). To examine male fertility, adult *Gdpd1*$^{-20/-20}$ males were mated with wild-type females for several months. *Gdpd1*$^{-20/-20}$ males were fertile, showing normal mating behavior with successful ejaculation and vaginal plug formation. The mean litter size was 9.0 ± 1.9 (total litter number: 6). Moreover, *Gdpd1* was expressed in not only testis but also ovary (Figure S7A). Even *Gdpd1* mutant females were fertile (mean litter size: 9.0 ± 1.0, total litter number: 5).

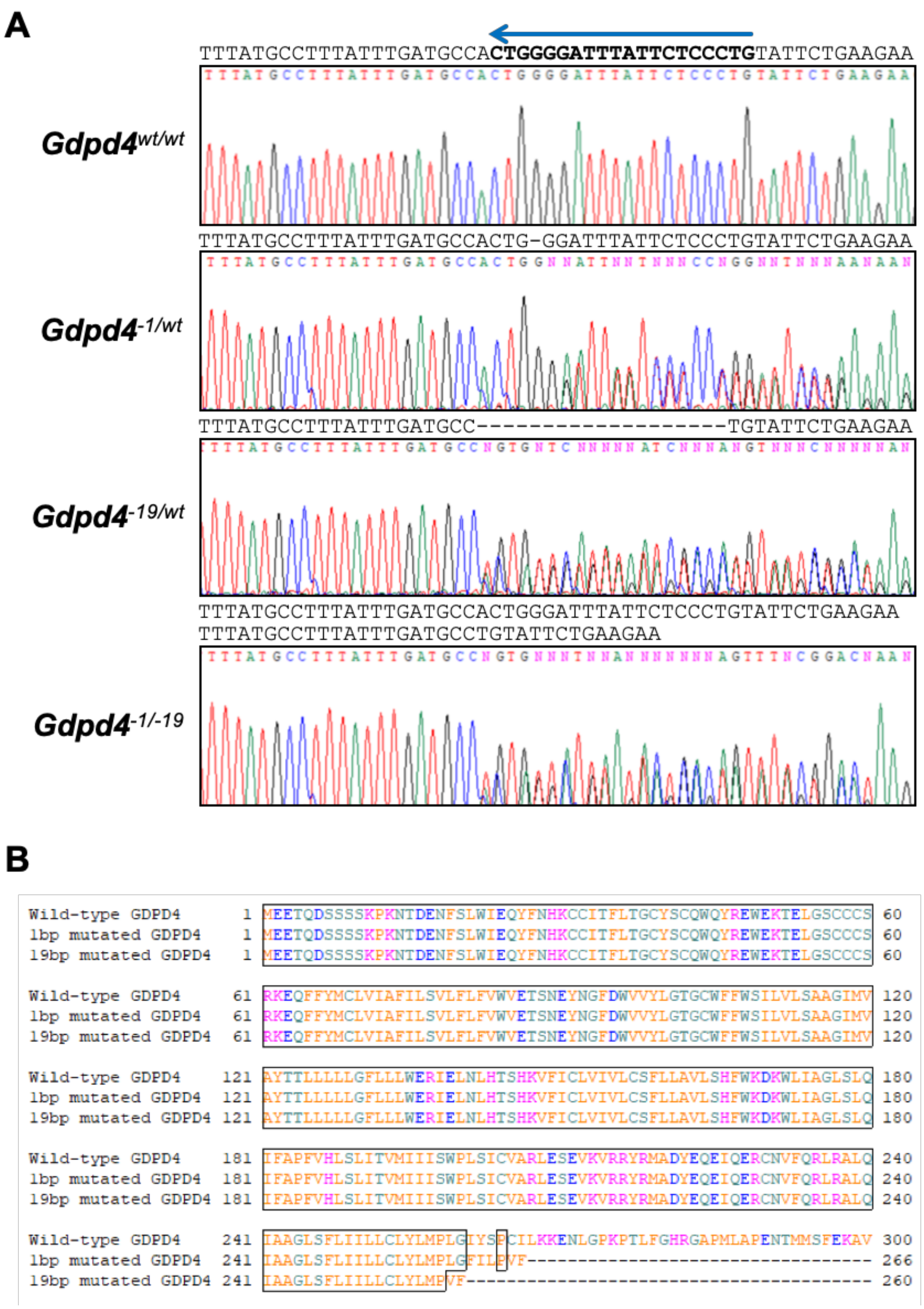

Fig. S8. Generation of *Gdpd4* mutant mice with CRISPR/Cas9. (A) Waveforms of direct sequencing of the 1 bp and 19 bp deletions [(5'-G-3') and (5'-ACTGGGGATTTATTCTCCC-3')] in *Gdpd4* gene. Arrow indicates gRNA sequence. (B) Amino acid sequence of *Gdpd1* mutant mice. The 1 bp and 19 bp deletions caused a frameshift mutation leading to a premature termination codon after the amino acid 266th and 260th, respectively (632 amino acids in wild-type mice). To examine male fertility, adult *Gdpd4*$^{-1/-19}$ males were mated with wild-type females for several months. *Gdpd4*$^{-1/-19}$ males were fertile, showing normal mating behavior with successful ejaculation and vaginal plug formation. The mean litter size was 9.0 ± 1.1 (total litter number: 11).

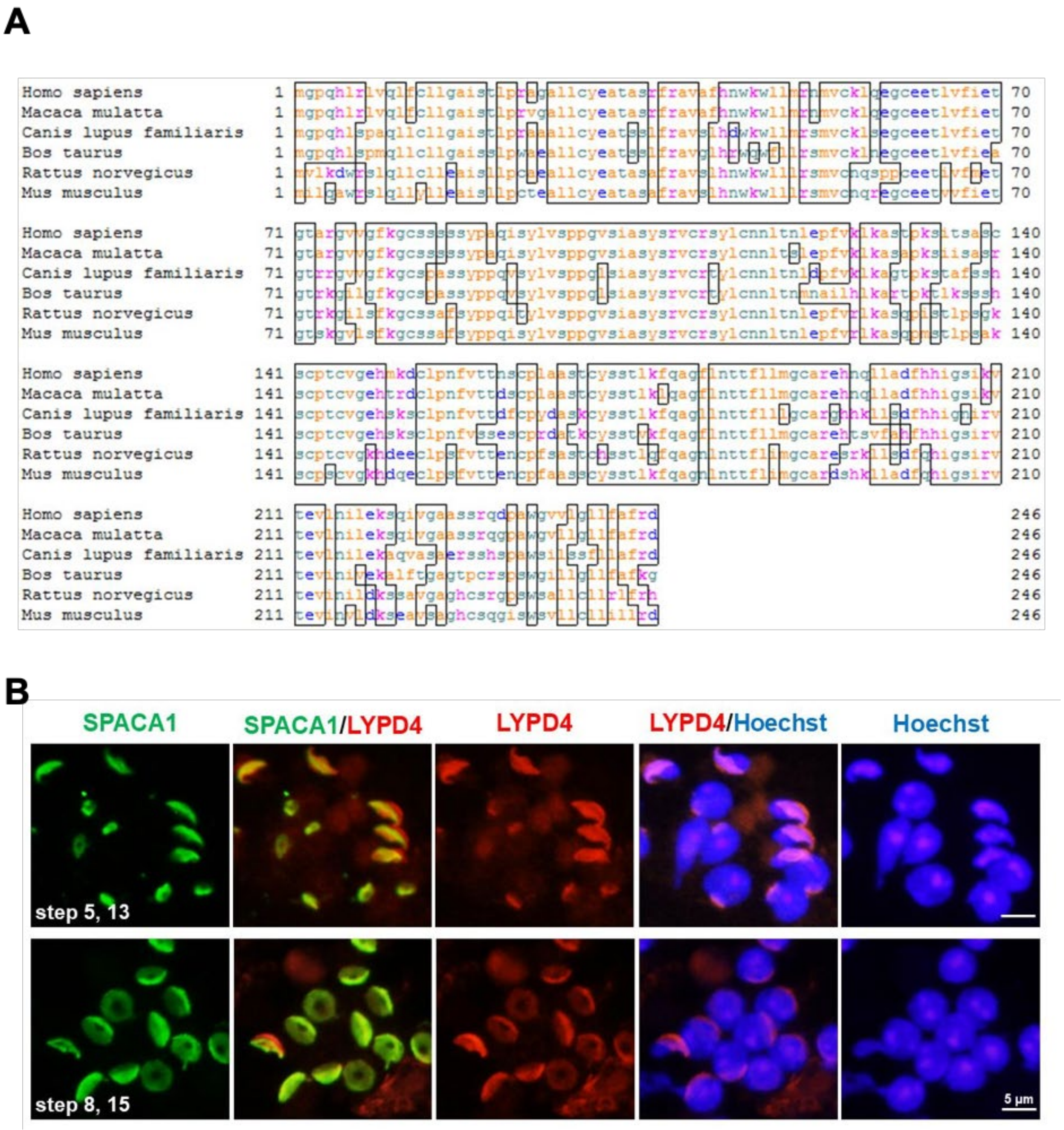

Fig. S9. Characterization of LYPD4 in mice. (A) Amino acid sequence similarity of LYPD4 among mammals (Homo sapiens, Macaca mulatta, Canis lupus familiaris, Rattus norvegicus, and Mus musculus). Box indicates a match in all sequences. LYPD4 is conserved broadly in mammals. (B) Immunostaining of LYPD4 in testicular germ cells. LYPD4, red signals, localized to acrosomal membrane. SPACA1, green signals, was used as a marker of sperm acrosomal membrane. Blue signals indicate nuclei stained with Hoechst 33342. Although LYPD4 and SPACA1 are co-localized at early stage of spermiogenesis (around step 8), each of them is localized to outer and inner acrosomal membrane at late stage of spermiogenesis. Scale bars: 5 µm.

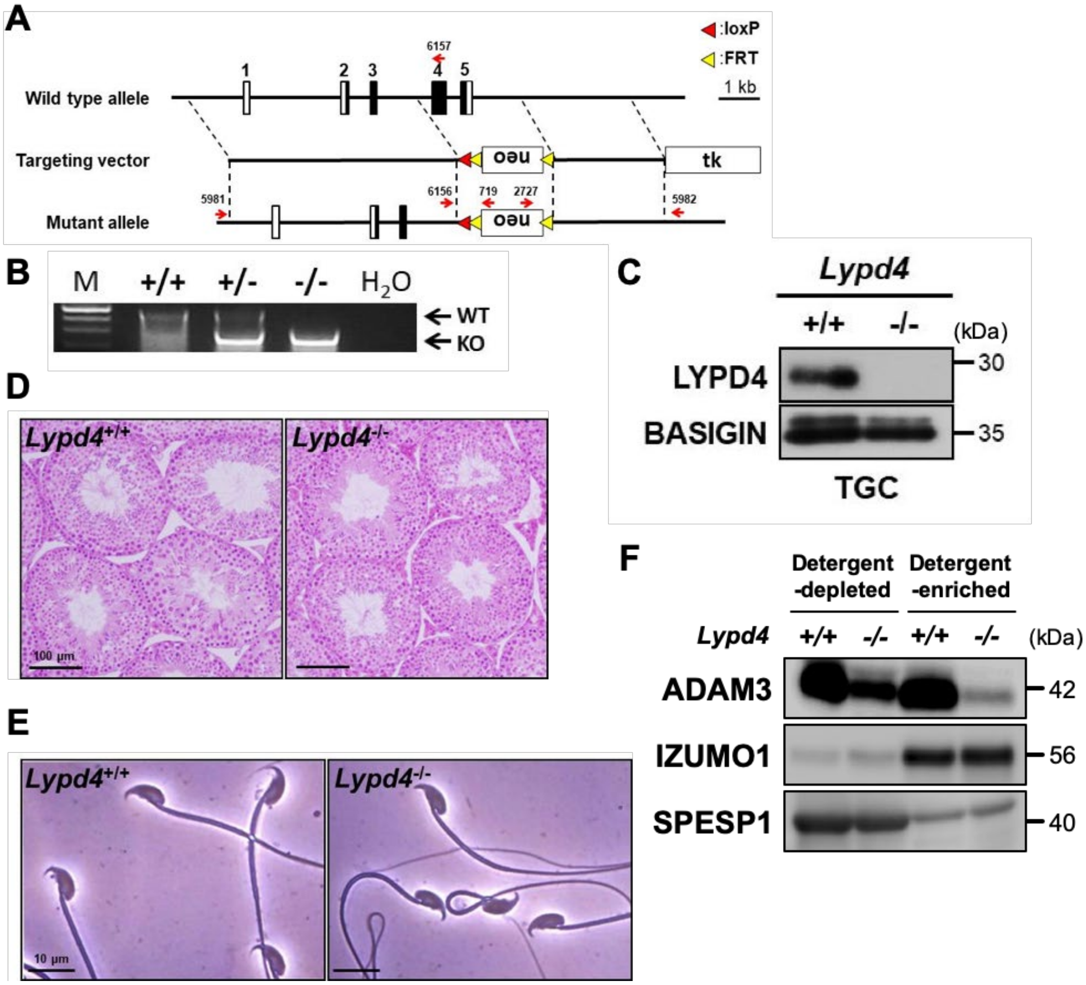

Fig. S10. Analysis of *Lypd4* KO mice. (A) Targeted disruption of *Lypd4* gene. *Lypd4* exons 4-5 are replaced with a floxed promoter-driven neo cassette. (B) Genotyping with PCR in *Lypd4* KO mice. Both a 496-bp band as the wild-type (+/+) allele and a 295-bp band as the knockout (-/-) allele were amplified by PCR. (C) Immunoblot analysis of LYPD4 using TGC lysates from *Lypd4* KO mice. BASIGIN was used as a loading control. (D) Representative testicular histology sections stained with hematoxylin and eosin. Spermatogenesis in *Lypd4* KO mice looked normal compared with that in wild-type mice. Scale bars: 100 μm. (E) Cauda epididymal spermatozoa from wild-type and *Lypd4* KO mice. Scale bars: 10 μm. (F) Triton X-114 extracts of cauda epididymal spermatozoa were examined after separation into detergent-depleted and detergent-enriched fractions. Although ADAM3 was detected in both the detergent-depleted and detergent-enriched phases of wild-type and *Lypd4* KO spermatozoa, the bands in both phases of *Lypd4* KO spermatozoa were weaker than that of wild-type spermatozoa. Immunoblot analysis using Triton X-100 extracts of cauda epididymal spermatozoa also showed that the amount of ADAM3 in *Lypd4* KO mice decreased compared to wild-type mice (Figure 7B). IZUMO1 (which contains a transmembrane domain) and SPESP1 (which localizes to the sperm equatorial segment) were used as control proteins, and they were distributed in the detergent-enriched and detergent-depleted phases, respectively.

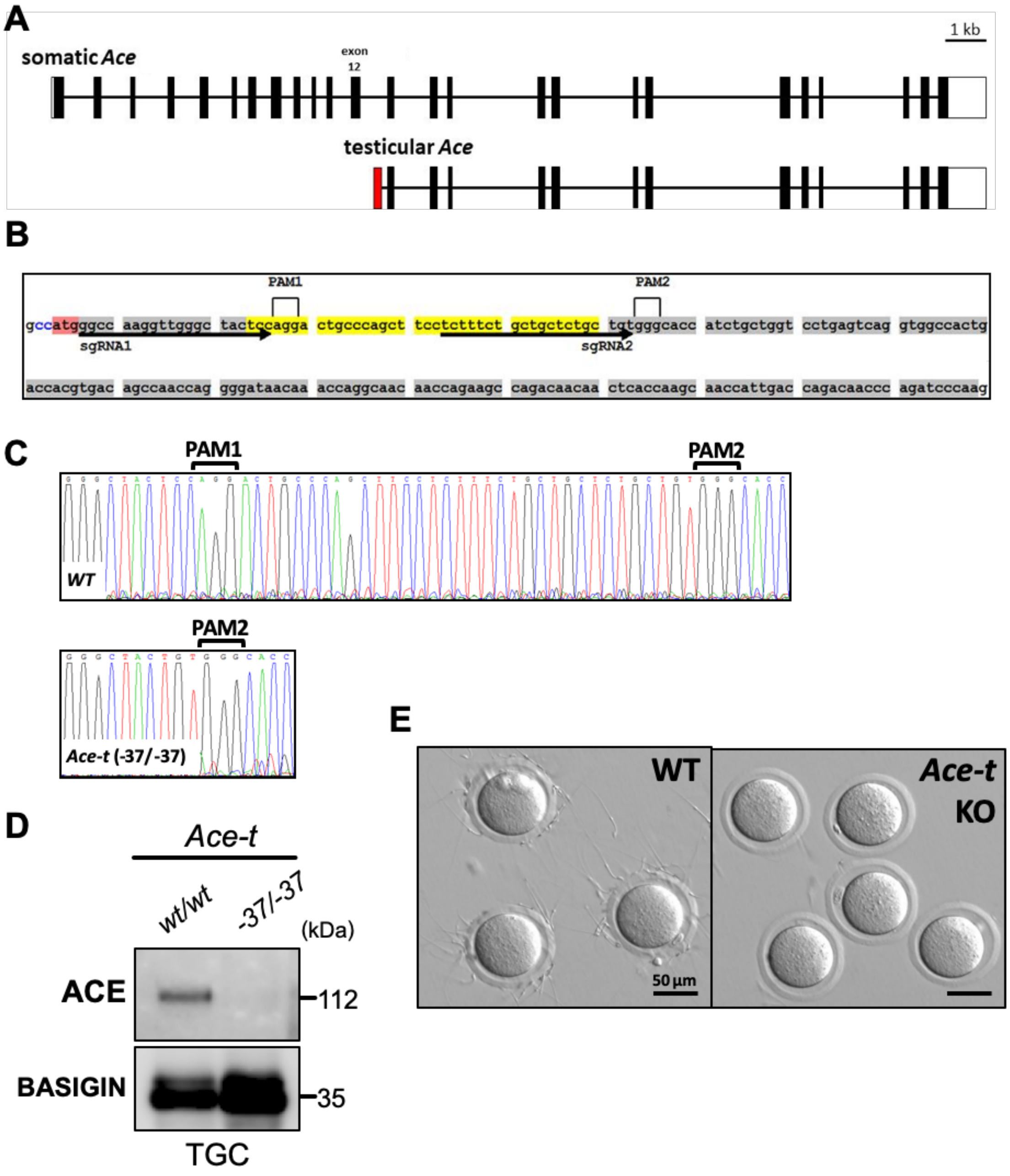

Fig. S11. Generation of testicular *Ace* (*Ace-t*) KO mice. (A) Exon-intron structure of *Ace* gene. There are two isoforms of ACE, somatic and testicular types. Somatic *Ace* (*Ace-s*) mRNA consists of all 26 exons except the 13th exon which is spliced, and testicular *Ace* (*Ace-t*) mRNA is used exons 13 to 26. Thus, 13th exon (red box) is expressed only in the testis. Scale bar: 1 kb. (B) Targeting scheme of the 37 bp deletion in the mouse *Ace* locus. Gray indicates coding region of 13th exon (red: start codon). Yellow indicates sequence of 37 bp deletion. Arrows indicate gRNA sequences (#1 and #2). (C) Waveforms of direct sequencing of the 37 bp deletion (5'-CTCCAGGACTGCCCAGCTTCCTCTTTCTGCTGCTCTG-3') in *Ace* gene. PAM (protospacer adjacent motif) sequence serves as a binding signal for CAS9. (D) Immunoblot analysis of ACE using TGC lysates from *Ace-t*$^{-37/-37}$ (KO) mice. BASIGIN was used as a loading control. (E) Observation of ZP-binding in wild-type and *Ace-t* KO spermatozoa. *Ace-t* KO spermatozoa had an impaired ZP-binding ability *in vitro* as reported previously. Scale bars: 50 µm.

Table S1. RNA-seq RPKM values of genes expressed in multiple tissues.

	#GeneID	Frontal Lobe	Genital fat pad	Heart	Kidney	Large Intestine	Liver	Lung	Ovary	Placenta	Small intestine	Spleen	Stomach	Testis	Thymus
Pate1	100312987	0.00	29.30	0.00	0.00	0.00	0.00	0.00	0.00	0.00	0.00	0.00	0.00	0.81	0.00
Pate2	330921	0.10	12.20	0.00	0.00	0.00	0.00	0.00	0.01	0.03	0.00	0.00	0.00	0.32	0.01
Pate3	100312956	0.03	12.50	0.00	0.00	0.00	0.00	0.00	0.00	0.01	0.00	0.00	0.00	0.12	0.00
Pate4	56872	0.03	0.58	0.00	0.00	0.00	0.00	0.00	0.00	0.03	0.00	0.00	0.00	0.07	0.01
Pate5	77080	0.01	137.00	0.00	0.00	0.01	0.00	0.00	0.00	0.00	0.00	0.00	0.04	0.00	0.00
Pate6	68171	0.01	74.50	0.00	0.00	0.04	0.00	0.00	0.00	0.00	0.00	0.00	0.03	0.72	0.00
Pate7	100312986	0.00	39.90	0.00	0.00	0.00	0.00	0.00	0.00	0.00	0.00	0.00	0.02	0.22	0.00
Pate8															
Pate9	434396	0.00	0.08	0.00	0.00	0.00	0.00	0.00	0.00	0.00	0.00	0.00	0.00	0.00	0.00
Pate10	100312949	0.00	15.60	0.00	0.00	0.00	0.00	0.00	0.00	0.00	0.00	0.00	0.01	0.00	0.00
Pate11	671003	0.04	0.00	0.00	0.00	0.00	0.00	0.00	0.00	7.18	0.00	0.00	0.00	0.00	0.00
Pate12	639025	0.01	0.01	0.00	0.00	0.00	0.00	0.00	0.00	5.21	0.00	0.00	0.00	0.01	0.06
Pate13	77908	0.00	36.40	0.00	0.00	0.00	0.00	0.00	0.00	0.00	0.00	0.00	0.01	0.01	0.00
Pate14	235973	0.01	0.27	0.00	0.00	0.00	0.00	0.00	0.03	0.08	0.00	0.00	0.00	0.79	0.09
Gm5916	546123	0.00	18.10	0.00	0.00	0.00	0.00	0.00	0.00	0.00	0.00	0.00	0.00	0.00	0.00
Gm27235															
Cst3	13010	1070.00	574.00	529.00	354.00	166.00	157.00	754.00	349.00	210.00	120.00	311.00	378.00	165.00	311.00
Cst8	13012	0.00	110.00	0.00	0.01	0.01	0.00	0.73	1.86	0.00	0.00	0.00	0.05	50.10	0.07
Cst9	13013	0.01	0.47	0.33	0.01	0.00	0.00	0.26	0.01	0.01	0.00	0.00	0.00	86.60	0.07
Cst11	78240	0.02	228.00	0.00	0.00	0.02	0.00	0.00	0.01	0.00	0.00	0.00	0.03	0.01	0.00
Cst12	69362	0.19	58.20	0.07	0.04	0.01	0.00	0.00	0.58	0.00	0.00	0.00	0.03	44.90	0.00
Cst13	69294	0.00	0.13	0.00	0.00	0.00	0.00	0.10	0.00	0.00	0.00	0.00	0.00	25.60	0.00
Cstdc1	78609	0.00	0.17	0.00	0.00	0.00	0.00	0.05	0.00	0.01	0.00	0.00	0.00	5.82	0.00
Cstdc2	77705	0.01	263.00	0.10	0.00	0.02	0.00	4.06	0.10	0.00	0.00	0.00	0.09	2.59	0.02
Cstl1	228756	0.17	0.11	0.01	0.00	0.00	0.00	0.01	0.01	0.01	0.00	0.00	0.00	37.10	0.00
Gdpd1	66569	9.26	2.36	3.15	0.57	61.40	0.11	5.82	4.18	0.87	15.50	0.83	3.31	41.00	1.83
Gdpd2	71584	1.80	0.45	0.11	0.31	8.05	0.00	4.31	14.60	0.07	13.20	33.60	2.71	0.39	0.42
Gdpd3	68616	0.66	5.49	4.94	15.00	8.38	2.24	3.91	3.04	0.77	2.00	5.96	5.17	0.46	5.68
Gdpd4	233537	0.90	0.02	0.00	0.00	0.00	0.00	0.00	0.00	0.00	0.00	0.00	0.00	4.57	0.00
Gdpd5	233552	11.10	5.61	7.65	3.07	6.12	0.26	8.96	14.10	4.16	5.66	4.82	8.46	3.74	3.22
Lypd1	72585	3.98	0.67	0.17	0.26	1.33	0.36	0.38	0.83	0.42	2.50	0.03	1.25	0.95	0.33
Lypd2	68311	0.16	0.02	8.28	10.00	0.00	0.02	49.00	0.01	0.00	0.02	0.00	1.81	0.05	2.06
Lypd3	72434	0.00	1.09	0.01	0.15	0.09	0.00	13.30	1.40	0.10	0.22	0.03	104.00	0.03	1.41
Lypd4	232973	0.00	0.69	0.13	0.07	0.00	0.00	0.00	0.00	0.01	0.00	0.00	0.00	398.00	0.02
Lypd5	76942	0.01	0.02	0.00	0.00	0.00	0.00	13.20	0.00	0.00	0.03	0.00	98.30	2.16	0.63
Lypd6	320343	2.96	0.72	0.09	1.18	0.14	0.01	0.45	0.69	0.44	0.17	0.12	1.80	0.14	0.09
Lypd6b	71897	1.29	0.26	0.03	0.45	0.09	0.00	0.40	0.62	0.10	0.18	0.17	1.77	0.05	1.34
Lypd8	70163	0.05	818.00	0.19	0.10	2860.00	1.43	9.52	0.39	4.50	883.00	0.16	758.00	2.10	8.71

Table S2. List of primers.

Figure	Sequence (5' to 3')	Name
1B	TCCCAATTACTGAGATGCCT	*Pate1*
	TTCATTGCACATGTCTTGGC	
	TGATCTGTCTGTTCTGCCAA	*Pate2*
	GCTCTGTCCTCCTTTCAAAA	
	TTGCTGCTCTTCTCCCTCTT	*Pate3*
	GTTACAAAAATCAGAGTTGC	
	ATGAATTCAGTGACGAAAATCAGCACACTG	*Pate4*
	CTAGAAGCTATTACACAAGTTTTTTTCGCAGC	
	TGCTCCTACACTTTCTGTTG	*Pate5 (9230110F15Rik)*
	ATTCACTACTTGGCCATGAT	
	AGGCTGTGTCTCTTTCTTCT	*Pate6 (D730048I06Rik)*
	TGTCATTACAGAAATCTCCA	
	AAAAGTACAGGAGCTGAGCA	*Pate7 (Gm17727)*
	TTCATCACTTGGCACAGCAA	
	TCTCTGCTCCTAGGATCCCA	*Pate8 (Gm17689)*
	CTGGTTACACAAAGAACTGG	
	GAATCCGGTGACAAAAATCA	*Pate9 (Gm5615)*
	GCAGAAAGATTTGTCATTGC	
	GTCTCATTCAATGGGGGAAC	*Pate10 (Gm17677)*
	TAGCTTTCATTGCAGCAGGA	
	ATGGGAAAGCACGTCTTGCA	*Pate11 (Gm9513)*
	GCAGAAAGATTTGTTGTTGC	
	ATGGGAAAGCACATCTTGCA	*Pate12 (Gm7257)*
	GCAGAAAGACCTTTTCTTGC	
	CATTGTAATCTGTGTTCGCA	*Pate13 (9230113P08Rik)*
	TCAATCATCGTTCAACTCTT	
	GTCCTGAGTACTCTGCCCTG	*Pate14 (A630095E13Rik*
	ATTATTTGTAGCTTCACAGG	
	ATCCTGCTCCTACTCTTTCT	*Gm5916*
	CACCTTTGCAACAAAAGGTA	
	AACTTCCTGAGGCTGTGTCT	*Gm27235*
	GATAACCTGGCAATTTTCCA	
	CATCCGTAAAGACCTCTATGCCAAC	*Actb*
	ATGGAGCCACCGATCCACA	
1C, 1D, 1E	AGCCAGCCTCTAGTGGAGACA	*Pate8* genotyping (Fw2)
	AAAAAAAGGGAGGGGGAATGA	*Pate8* genotyping (Rv2)
	CCCTTGAACCTGGGATTGTTT	*Pate10* genotyping (Fw1)
	TTTGTATCACCCTCCCCACCT	*Pate10* genotyping (Rv1)
3B	GCTCCTTGCTGTTCCTGCTG	*Cst3*
	GCTGGTCATGGAAAGGACAGTC	
	TTGTGGCTCTCCTTGATCCTC	*Cst8*
	TGCAATTACTGCGGGAGATC	
	AGAAAGCTCTGCCTCTCACCA	*Cst9*
	ATTCAGACCATGGCTCTCCTG	
	ATCTTGGAAGGCAACACGG	*Cst11*
	TGTGAAGTTCCCCTTTCTGG	
	CGTGGCACTCATTGTGCTG	*Cst12*
	TTCTCTCTCCTGGACCTTCCT	
	TTCCTGGTGATCATGGTGGA	*Cst13*
	CTCCTGCAATCTTCTTGCAC	
	TAGGACTGGTTGTGCTAGGCA	*Cstdc1*
	TTGAGCCTTGTAGCAATGGG	
	TGGTATCTATGTCCAGGGAGC	*Cstdc2*
	CGGTGAAAGGGTGGATACTCA	
	AAGGCCAGAGGTTTGAGGAT	*Cstl1*
	TCAGCTTGCTGCTTTGCA	
	CATCCGTAAAGACCTCTATGCCAAC	*Actb*
	ATGGAGCCACCGATCCACA	
3C, 3D, 3E	CTCTGAAGTCTGTTCTTGGG	*Cstl1* genotyping (Fw3)
	GTAGGTGTCGTTGCTTGCGT	*Cstl1* genotyping (Rv3)
	CATTCCAAGGAAAGTGACCCA	*Cstdc2* genotyping (Fw4)
	TGATATCGGGGCAAGTACTG	*Cstdc2* genotyping (Rv4)

Table S3. List of primers (continued).

5A	ATGTGTCAGAAAGAAGTGATGGAGCAAAGTG	*Lypd1*
	TCAGCAGTGTGCCAAGCAGAGG	
	CTATGGCCTTACAGTGCTACACCTGTG	*Lypd2*
	TTACAGCAGGACACCCAAGAGCAAG	
	GGCCGTGTCTACAAGGGCTGC	*Lypd3*
	TCACAGCATCGCGCCAGCAAC	
	GAGACTCAAGGCCAGTCAACCCATG	*Lypd4*
	TCAATCTCTGAGGAGTATAAGAAGGCATAAGAG	
	CCTGAGAAGTCCCGACGGGTCC	*Lypd5*
	CTATGCAGGGAACCCGACGGAC	
	CACCAGATACTGCTACACTCAGCACAC	*Lypd6*
	TTATAAGGTGAGTCCCAGCCACACCC	
	CAGAAGACAAGTGGTGTCCGCAGG	*Lypd6b*
	TCATAGCAAGGGAAGCATGAAGAGCC	
	GGAGAAACCCCAAAAGTGTTACGAAGGAG	*Lypd8*
	TCAGAACAGCAGCTTCAGAAGAAGAAGG	
	AAGTGTGACGTTGACATCCG	*Actb*
	GATCCACATCTGCTGGAAGG	
5B	GTCTATCACATCTGCGTCCTG	Human *LYPD4*
	ACCTCAGTCACTTTGATGCTC	
	AATCCCATCACCATCTTCCAG	Human *GAPDH*
	ATGACCCTTTTGGCTCCC	
S1B, S1C, S1D	AAATAAAGTCTTTTGTTTGT	*Pate2* genotyping (Rv2)
	GTTATCATACATGGTGTGTC	*Pate3* genotyping (Fw1)
	TTTCACATCTTAATTACTGT	*Pate3* genotyping (Rv1)
S7A	GCGAGAACATCTGACAGTGTGGGG	*Gdpd1*
	CTTAAGCTTGGTTGGGTAGTCCGTCATC	
	GCGCCAGATATATGGACATCAGGGAG	*Gdpd2*
	TCACTCAGAGGCGAAATTGTTGATCTTGG	
	CCCATGGCCTTCACGATATGGCG	*Gdpd3*
	GACAGGACAGGGCCTCAGGC	
	CTTGGCTCTTCTCACTGGCCTGG	*Gdpd4*
	CAGGGGCTAAGTTGGCAGACGTAG	
	CCTCCGTCACTTCCGACAACTCCC	*Gdpd5*
	CTAATGCCCACTCTGCTCTGTGACG	
	AAGTGTGACGTTGACATCCG	*Actb*
	GATCCACATCTGCTGGAAGG	
S7B	CCAACCTCTTGCCTCGGGGTC	*Gdpd1* genotyping
	CCTCCTCACAGAAGCCCTGTCAC	
S8A	CAGTGGTAGAGCACTTGCCTGGC	*Gdpd4* genotyping
	CCCTATGCCTTAGAGTCGTTCCTCCC	
S10A	GCCTTCTATCGCCTTCTTGACGAGTTCTTC	*Lypd4* 5'-arm screening (#719/#5981)
	CTCAAGCACGCTCACACTCTGGTCTAC	
	CAGCCTCTGAGCCCAGAAAGCG	*Lypd4* 3'-arm screening (#2727/#5982)
	CACTTAACCATGCAACCACACCTATCATC	
S10B	ACAGCTCACTCCATTGCATTTGCC	*Lypd4* genotyping
	CTGGTTAGCACACAAGCAAAGGGG	
	ATCCGGGGGTACCGCGTCGAG	
S11C	GGGTATGTCTTTCTCTCAGGGTCCTAGAG	*Ace-t* genotyping
	GCCTTGGCTTCATCAGTCTCTAGGT	

References

1. F. Yue *et al.*, A comparative encyclopedia of DNA elements in the mouse genome. *Nature* **515**, 355-364 (2014).
2. T. Noda *et al.*, Nine genes abundantly expressed in the epididymis are not essential for male fecundity in mice. *Andrology* (2019).
3. S. G. Goodson, Z. Zhang, J. K. Tsuruta, W. Wang, D. A. O'Brien, Classification of mouse sperm motility patterns using an automated multiclass support vector machines model. *Biol Reprod* **84**, 1207-1215 (2011).
4. Y. Fujihara *et al.*, Expression of TEX101, regulated by ACE, is essential for the production of fertile mouse spermatozoa. *Proc Natl Acad Sci U S A* **110**, 8111-8116 (2013).
5. Y. Fujihara, A. Oji, K. Kojima-Kita, T. Larasati, M. Ikawa, Co-expression of sperm membrane proteins CMTM2A and CMTM2B is essential for ADAM3 localization and male fertility in mice. *J Cell Sci* **131** (2018).
6. Y. Fujihara, M. Okabe, M. Ikawa, GPI-anchored protein complex, LY6K/TEX101, is required for sperm migration into the oviduct and male fertility in mice. *Biol Reprod* **90**, 60 (2014).
7. K. Tokuhiro, M. Ikawa, A. M. Benham, M. Okabe, Protein disulfide isomerase homolog PDILT is required for quality control of sperm membrane protein ADAM3 and male fertility [corrected]. *Proc Natl Acad Sci U S A* **109**, 3850-3855 (2012).
8. Y. Fujihara *et al.*, SPACA1-deficient male mice are infertile with abnormally shaped sperm heads reminiscent of globozoospermia. *Development* **139**, 3583-3589 (2012).
9. Y. Fujihara *et al.*, Sperm equatorial segment protein 1, SPESP1, is required for fully fertile sperm in mouse. *J Cell Sci* **123**, 1531-1536 (2010).
10. Y. Fujihara, A. Oji, T. Larasati, K. Kojima-Kita, M. Ikawa, Human Globozoospermia-Related Gene Spata16 Is Required for Sperm Formation Revealed by CRISPR/Cas9-Mediated Mouse Models. *Int J Mol Sci* **18** (2017).
11. Y. Fujihara, M. Ikawa, CRISPR/Cas9-based genome editing in mice by single plasmid injection. *Methods Enzymol* **546**, 319-336 (2014).
12. D. Mashiko *et al.*, Generation of mutant mice by pronuclear injection of circular plasmid expressing Cas9 and single guided RNA. *Sci Rep* **3**, 3355 (2013).
13. Y. Naito, K. Hino, H. Bono, K. Ui-Tei, CRISPRdirect: software for designing CRISPR/Cas guide RNA with reduced off-target sites. *Bioinformatics* **31**, 1120-1123 (2015).
14. M. Muto, Y. Fujihara, T. Tobita, D. Kiyozumi, M. Ikawa, Lentiviral Vector-Mediated Complementation Restored Fetal Viability but Not Placental Hyperplasia in Plac1-Deficient Mice. *Biol Reprod* **94**, 6 (2016).
15. Y. Satouh, N. Inoue, M. Ikawa, M. Okabe, Visualization of the moment of mouse sperm-egg fusion and dynamic localization of IZUMO1. *J Cell Sci* **125**, 4985-4990 (2012).
16. Y. Fujihara, K. Kaseda, N. Inoue, M. Ikawa, M. Okabe, Production of mouse pups from germline transmission-failed knockout chimeras. *Transgenic Res* **22**, 195-200 (2013).
17. R. Yamaguchi, K. Yamagata, M. Ikawa, S. B. Moss, M. Okabe, Aberrant distribution of ADAM3 in sperm from both angiotensin-converting enzyme (Ace)- and calmegin (Clgn)-deficient mice. *Biol Reprod* **75**, 760-766 (2006).
18. A. Oji *et al.*, CRISPR/Cas9 mediated genome editing in ES cells and its application for chimeric analysis in mice. *Sci Rep* **6**, 31666 (2016).
